# Evolution of salivary glue genes in *Drosophila* species

**DOI:** 10.1186/s12862-019-1364-9

**Published:** 2019-01-29

**Authors:** Jean-Luc Da Lage, Gregg W. C. Thomas, Magalie Bonneau, Virginie Courtier-Orgogozo

**Affiliations:** 1 UMR 9191 Évolution, Génomes, Comportement, Écologie. CNRS, IRD, Université Paris-Sud. Université Paris-Saclay, F-91198 Gif-sur-Yvette, France; 2 Department of Biology and Department of Computer Science, Indiana University, Bloomington, IN 47405 USA; 3 Institut Jacques Monod-CNRS UMR7592-Université Paris Diderot, 15 rue Hélène Brion, 75013 Paris, France

**Keywords:** *Drosophila*, Glue, Internal repeats, Sgs, Pupa, Adaptation, Disordered protein, Eig71Ee, Mucin, Gene family, Gene copy number, Salivary gland

## Abstract

**Background:**

At the very end of the larval stage *Drosophila* expectorate a glue secreted by their salivary glands to attach themselves to a substrate while pupariating. The glue is a mixture of apparently unrelated proteins, some of which are highly glycosylated and possess internal repeats. Because species adhere to distinct substrates (i.e. leaves, wood, rotten fruits), glue genes are expected to evolve rapidly.

**Results:**

We used available genome sequences and PCR-sequencing of regions of interest to investigate the glue genes in 20 *Drosophila* species. We discovered a new gene in addition to the seven glue genes annotated in *D. melanogaster.* We also identified a phase 1 intron at a conserved position present in five of the eight glue genes of *D. melanogaster*, suggesting a common origin for those glue genes. A slightly significant rate of gene turnover was inferred. Both the number of repeats and the repeat sequence were found to diverge rapidly, even between closely related species. We also detected high repeat number variation at the intrapopulation level in *D. melanogaster*.

**Conclusion:**

Most conspicuous signs of accelerated evolution are found in the repeat regions of several glue genes.

**Electronic supplementary material:**

The online version of this article (10.1186/s12862-019-1364-9) contains supplementary material, which is available to authorized users.

## Background

Animals interact with their environment (viruses, bacteria, food, chemicals, conspecifics, etc.) in many different ways, particularly through their immune and sensory systems. As animals adapt to new places, the way they interact with their environment is expected to change. Accordingly, the gene families that have been shown to exhibit accelerated rates of gene gain and loss in several animal groups are mostly genes that mediate the interactions with the environment: immune defense, stress response, metabolism, cell signaling, reproduction and chemoreception [1]. Rapid changes in gene copy number can lead to fast phenotypic changes via gene deletion and can provide raw material for genes with new functions via gene duplication [2, 3]. Rapid turnover of genes within a gene family has also been shown to correlate with fast evolution at the sequence level [4, 5].

One particularly interesting environmental interaction occurs in *Drosophila*. Metamorphosis is a critical stage of fruitfly development [6] during which the animal is vulnerable and motionless. In Drosophilids pupae are generally attached to a substrate until the imago leaves the puparium. It is critical for the pupa to be firmly attached in order not to be moved away by some external event (i.e. rain or wind). Furthermore, for the emerging adult to be able to hold on the external substrate and thus get out of the pupal case, it is necessary for the pupa to adhere to a substrate, whether dry or wet. When the pupal case freely moves and is not attached, adults are unable to hatch and eventually die (J. R. David, personal communication).

Here we focus on the Salivary gland secretion (*Sgs*) genes, a functional group that mediates the physical interaction of flies in the genus *Drosophila* with an external substrate during metamorphosis. The *Sgs* genes encode proteins that make up the glue produced by *Drosophila* larvae that serves to attach the animal to a surface where it can undergo metamorphosis. In *D. melanogaster*, the glue is composed of several salivary gland secretion proteins which accumulate in the salivary glands of late third instar larvae [7]. As the puparium forms, the bloated salivary glands release their contents through the mouth. This secretion then hardens within seconds of contact with the air and becomes a glue which firmly attaches the pupa to the substrate.

Pupariation sites of *Drosophila* species in nature have not been extensively characterized, but a large variety of of pupariation sites have been found. In the wild, *D. melanogaster* pupae have been found adhered to wood, fixed to grape stalks, attached to the dry parts of various rotten fruits, or adhered to one another on the land beneath grape stalks [8–10]. *D. mauritiana* pupae may be found on the surface of decaying *Pandanus* fruit, which is hard and lignous (D. Legrand, personal communication). Many Hawaiian *Drosophila* species pupariate several inches deep in the soil [11]. Some other *Drosophila* species, such as *D. sechellia, D. simulans*, and the invasive *D. suzukii*, appear to pupariate directly within the wet rotten part of fruits (J. David, personal communication, [12]). Given the diversity of pupariation sites, we hypothesized that the different *Drosophila* species would require distinct types of glue meaning that the *Sgs* genes might evolve rapidly within the genus.

### The glue genes

The glue genes have long been an important model for the regulation of gene expression. In the 1970s and 1980s it was discovered that genes for proteins contained in salivary secretions correlate with the chromosomal location of major puffs. This led to the discovery that, on an acid-urea electrophoresis gel, the salivary glue was resolved into five major bands, numbered from 1 to 5 in order of increasing electrophoretic mobility [13, 14]. Band 2, which was variable and detected in many other tissues, was considered to be a tissue contamination rather than a true glue protein [13]. From this, seven glue genes were eventually identified, and their nucleotide sequences are now well characterized: *Sgs1* (band 1, *CG3047*, 2 L), *Sgs3* (band 3, *CG11720*, 3 L), *Sgs4* (band 4, *CG12181*, X), *Sgs5* (band 5, *CG7596*, 3R), *Sgs7* (*CG18087*, 3 L), and *Sgs8* (*CG6132*, 3 L) and *Eig71Ee* (also named *geneVII I71–7* or *gp150, CG7604*, 3 L) [15–25]. *Eig71Ee*, located at position 71E, is not only expressed in salivary glands but also in hemocytes and in the gut, where it appears to be involved in immunity and clotting [26–28].

A sixth electrophoretic band migrating slightly slower than the Sgs3 protein was also detected in a few *D. melanogaster* lines [14, 29, 30]. The nucleotide sequence of the corresponding gene, *Sgs6*, remains unknown but cytogenetic and genetic mapping indicates that *Sgs6* is located in region 71C3–4 and differs from *Eig71Ee* [21, 26, 30].

The three genes *Sgs3*, *Sgs7* and *Sgs8* form a tightly linked cluster on the 3 L chromosomal arm at position 68C [31, 32]. All glue genes were found to start with a signal peptide. The largest glue genes, *Sgs1*, *Sgs3* and *Sgs4* and *Eig71Ee* were shown to harbor numerous internal repeats of amino acid motifs, rich in proline, threonine and serine [16, 23, 27, 33]. Molecular studies showed that the number of internal repeats was variable between strains in Sgs3 [34], and Sgs4 [33]. Additionally, consistent with missing protein bands, a few laboratory strains were inferred to carry loss-of-function mutations in *Sgs4* [7, 13, 33, 35], *Sgs5* [25] and *Sgs6* [14, 29, 30].

In the present study, we characterize the diversity and evolution of the *Sgs* genes within the *Drosophila* genus. We inferred loss and gain of glue genes and we investigated repeat number variation and sequence repeat diversity across 19 species and across paralogs.

## Results

**Fig. 1:**
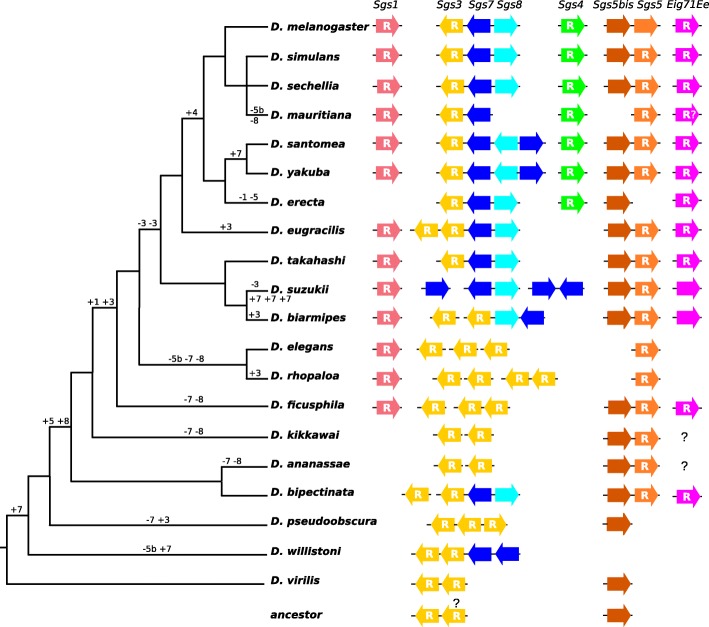
Schematic species tree showing glue gene distribution and the most parsimonious scenario for gene gains and losses. Gains are indicated by “+” and losses by “-”. Numbers correspond to the glue gene name (eg. “3” for Sgs3). An inferred distribution of glue genes in the last common ancestor is shown at the bottom. The tree is from Thomas, G.W.C. and Hahn M.W. (2017) 10.6084/m9.figshare.5450602. Pink is for *Sgs1*, yellow is for *Sgs3*, dark blue is for *Sgs7*, light blue is for *Sgs8*, green is for *Sgs4*, orange is for *Sgs5-5bis*, purple is for *Eig71Ee*. Along with each species is a schematic representation of the organization of the glue gene cluster, with relative position and orientation for the species with confirmed synteny information. Gene sizes and distances are not to scale. “R” means that internal repeats are present. “R?” means that no clear repeats were identified. In *D. pseudoobscura*, the relative orientation of the three clustered *Sgs3*-like sequences GA25425, GA23426, GA23878 suggested that GA23426 could be orthologous to *Sgs3* (it is inside an intron of GA11155, homologue of Mob2, which is close to *Sgs3* in *D. melanogaster*), GA23425 to *Sgs7* and GA23878 to *Sgs8*. The last two had more similar sequences compared to GA23426, including the repeat region. Furthermore, the latter was neighbor to GA20420, a homologue of *chrb-PC*, a gene adjacent to *Sgs8* in *D. melanogaster*

We used the six *Sgs* genes and *Eig71Ee* annotated in *D. melanogaster* as BLAST queries to identify their putative homologs in 19 other *Drosophila* species (Table 1). The homologs are summarized in Fig. 1 and Table 2. In *D. melanogaster*, the glue genes are “extremely highly” or “very highly” expressed in late larval salivary glands according to the RNAseq data in Flybase. But transcript data that would be useful for annotating the genes were not available for all species, probably because the expression window of the glue genes (late third larval instar and only in salivary glands) is narrow [7]. The organization of the *Sgs* genes was found to be generally conserved across the *Drosophila* species we investigated (Fig. 1). Proper identification of each ortholog was based on sequence similarity and, when possible, synteny. We describe below our findings for each category of *Sgs* genes.

**Table 1:** List of species and databases used in this study

Species	Database	Version	URL	Date of access	reference
*melanogaster*	FlyBase	FB2015_02	flybase.org	06/2016	[60]
*simulans*	FlyBase	FB2015_02	flybase.org	02/2017	[60]
*sechellia*	FlyBase	FB2015_02	flybase.org	02/2017	[60]
*mauritiana*		v1.0	www.popoolation.at/mauritiana_genome/	12/2016	[63]
*yakuba*	FlyBase	FB2015_02	flybase.org	02/2017	[60]
*santomea*		v1.0	genomics.princeton.edu/AndolfattoLab/Dsantomea_genome.html	11/2016	[62]
*erecta*	FlyBase	FB2015_02	flybase.org	02/2017	[60]
*takahashii*	FlyBase	FB2015_02	flybase.org	02/2017	[60]
*ficusphila*	FlyBase	FB2015_02	flybase.org	02/2017	[60]
*biarmipes*	FlyBase	FB2015_02	flybase.org	02/2017	[60]
*suzukii*	SpottingWingFlybase	v1	http://spottedwingflybase.org/	02/2017	[61]
*eugracilis*	FlyBase	FB2015_02	flybase.org	02/2017	[60]
*elegans*	FlyBase	FB2015_02	flybase.org	02/2017	[60]
*rhopaloa*	FlyBase	FB2015_02	flybase.org	02/2017	[60]
*kikkawai*	FlyBase	FB2015_02	flybase.org	02/2017	[60]
*ananassae*	FlyBase	FB2015_02	flybase.org	02/2017	[60]
*bipectinata*	FlyBase	FB2015_02	flybase.org	02/2017	[60]
*willistoni*	FlyBase	FB2015_02	flybase.org	02/2017	[60]

**Table 2:** Genomic coordinates of the glue genes in 20 *Drosophila* species

Species	*Sgs1*	*Sgs3*	*Sgs4*	Sgs5 Sgs5bis*	*Sgs7*	*Sgs8*	*Eig71Ee*
*D. melanogaster*	CG3047	CG11720	CG12181	CG7596 CG7587*	CG18087	CG6132	CG7604
*D. simulans*	GB:CM002910 4,752,550–4,754,973	Dsim\GD14311	Dsim\GD16637	Dsim\GD19170 Dsim\GD19169*	Dsim\GD17634	Dsim\GD28639	Dsim\GD12546
*D. sechellia*	Dsec\GM18501 (M)	Dsec\GM25279 (M)	GB:CH480825 2,852,711–2,853,386 (M)	Dsec\GM15245 Dsec\GM15244*	Dsec\GM25278	Dsec\GM24748	NW_001999689 7,761,215–7,759,941
*D. mauritiana*	2 L: 4721427–4,722,731	3 L: 11002313–11,003,109	X: 2864998–2,865,616 (M)	3R: 7695225–7,694,660 relictual Sgs5bis 3R: 7696600–7,695,629	3 L: 10999955–11,000,249	no	3 L: 15018149–15,017,249
*D. yakuba*	NT_167062 10,588,365–10,585,585	Dyak\Sgs3	Dyak\GE28681	Dyak\GE25481 Dyak\GE25480*	Dyak\GE20214 Dyak\GE21218	Dyak\Sgs8	Dyak\GE19823
*D. santomea*	2 L: 10595909–10,588,129	3 L: 11541799–11,542,678 (M)	X: 5242740–5,241,688 (M)	3R: 1975190–1,975,883 3R: 1974195–1,974,756*	3 L: 11539572–11,539,861 3 L: 11536774–11,536,485	3 L: 11537383–11,537,681	3 L: 18202978–18,201,736
*D. erecta*	no	Dere\Sgs3	Dere\GG27095	no Sgs5 Dere\GG22329*	Dere\GG13918	Dere\Sgs8	Dere\GG13528
*D. eugracilis*	AFPQ02004874 817,906–819,883	KB465257 3,401,691–3,402,412 3,385,186–3,386,300	no	KB464468 62,658–63,338 61,657–62,202*	KB465257 3,378,701–3,378,995	KB465257 3,378,110–3,377,822	KB464880 383,836–382,228 (XM_017230731)
*D. takahashii*	KB461520 248,469–250,276	KB460792 317,161–317,949	no	KB461611 188,299–187,637 189,545–188,599*	KB461234 120,246–120,467	KB461234 119,117–118,896	XM_017142344
*D. ficusphila*	KB457325 1,315,471–1,313,145	KB457563 3,180,441–3,179,541 KB457373 332,100–331,262 3,199,436–3,198,351	no	KB457381 2,059,719–2,058,971 2,061,615–2,060,148*	no	no	KB457515 1,660,700–1,661,809 (XM_017197540)
*D. biarmipes*	KB462641 1,521,394–1,523,538	KB462590 1,536,842–1,537,624 (M) KB462646 54,238–53,374 (M)	no	KB462814 8,082,338–8,083,047 8,081,336–8,081,891*	KB462646 76,095–75,801	KB462646 77,216–77,501	KB462754 733,209–734,564
*D. suzukii*	KI419149 6,645,021–6,638,237	no	no	KI420542 10,372–9639 11,441–10,912*	KI419359 22,757–22,464 KI420769 54,293–54,584 KI420610 25,121–25,412 55,385–55,094	KI420769 53,260–52,976	XM_017082231
*D. elegans*	KB458429 2,603,084–2,605,600	KB458268 2,467,758–2,468,497 KB458387 820,622–819,957 KB458387 18,429–17,499	no	KB458458 2,864,199–2,863,401 no Sgs5bis	no	no	no
*D. rhopaloa*	KB450401 (Nterm) KB452165 (Cterm)	KB450817 117,692–118,515 KB452471 215,593–216,424 KB451944**	no	KB451039 15,186–16,018 no Sgs5bis	no	no	no
*D. kikkawai*	no	KB459615 1,331,679–1,331,220 KB459522 291,906–292,542	no	KB459676 1,112,222–1,111,011 1,113,233–1,112,671*	no	no	KB459876 1,106,397–1,107,027 (Nterm)
*D. ananassae*	no	NW_001939300 3,959,435–3,957,637 NW_001939293 5,806,878–5,808,646	no	NW_001939291 17,741,832–17,741,201 17,742,892–17,742,284*	no	no	GF10382(Nterm): NW_001939293 11,506,744–11,507,112
*D. bipectinata*	no	KB464001 557,673–558,039 KB464098 1,120,437–1,121,198	no	KB464382 185,749–186,362 184,743–185,354*	KB464098 1,109,828–1,110,127	KB464098 1,109,077–1,108,802	KB464259 2,466,431–2,466,234 (ortholog of GF10382)
*D. pseudoobscura*	no	GA23425, GA23426, GA23878	no	no Sgs5 Dpse\GA20459 *	no	no	no
*D. willistoni*	no	NW_002032853 3,296,683–3,295,766 NW_002032860 11,643,758–11,641,972	no	no	NW_002032853 2,792,051–2,792,347 2,793,811–2,794,107	no	no
*D. virilis*	no	NW_002014431 6,839,085–6,838,999 (GJ27025) 6,841,799–6,840,888(GJ26085)	no	no Sgs5 NW_002014424 14,511,533–14,512,083*(modified from GJ24445)	no	no	no

* indicates annotations and coordinates of the *Sgs5bis* gene; “M” indicates that part of the coding sequence was inferred manually by sequencing of PCR amplicons of relevant regions; “no” means that the gene sequence was not found by BLAST searches; Nterm and Cterm mean N-terminal and C-terminal region, respectively. **: this contig probably contains two paralogs of *Sgs3* with uncertain sequences

### Identification of a Sgs5 paralog

**Fig. 2:**
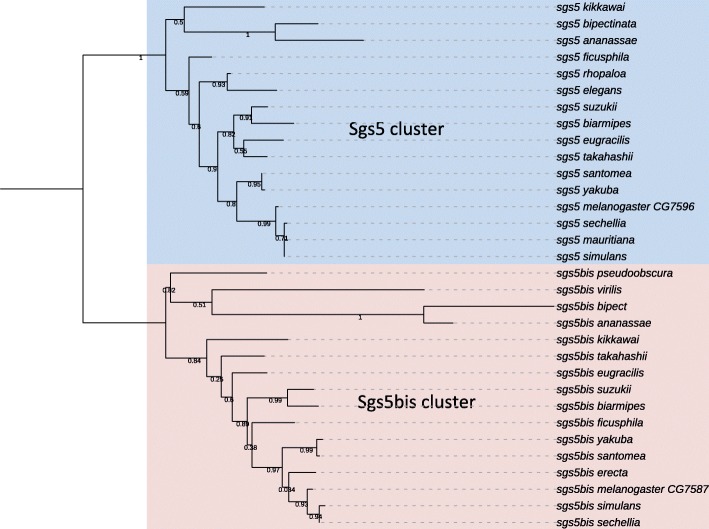
Maximum likelihood (ML) tree of aligned Sgs5 and Sgs5bis amino acid sequences (repeated parts removed when present). Numbers along branches are the posterior probabilities. The tree was rooted between the Sgs5 cluster and the Sgs5bis cluster

We found that *Sgs5* has a tandem paralog in *D. melanogaster*, located ca. 300 bp upstream of *Sgs5* (*CG7587*, hereafter named *Sgs5bis*), sharing 46,3% identity and 66,9% similarity at the protein level. Similar expression profiles from Gbrowse (flybase.org) and FlyAtlas (flymine.org) show that *Sgs5bis* is co-expressed with *Sgs5* during the late third larval instar in dissected salivary glands, and both genes harbor two introns in all species. To our knowledge, this paralog has not been mentioned earlier. The *Sgs5/5bis* pair is widely distributed in our species sample, and is therefore probably ancestral to most of the species studied. Occasional losses of either *Sgs5* or *Sgs5bis* occurred at least four times (Fig. 1): 1) loss of *Sgs5bis* in *D. mauritiana*, where a relictual sequence may still be recognized, 2) loss of *Sgs5bis* in *D. elegans,* 3) loss of *Sgs5bis* in *D. rhopaloa*, 4) loss of *Sgs5* in *D. erecta*. These patterns of loss suggest that *Sgs5* and *Sgs5bis* can replace each other functionally. There is no *Sgs5* nor *Sgs5bis* in *D. willistoni*. In *D. ananassae,* the orthologous sequence of *Sgs5bis* (formerly Dana\GF19880 in FlyBase release R1.3, with a different intron/exon structure) has been withdrawn from the genome annotation for reasons unknown to us though it is in conserved synteny relative to *D. melanogaster.* In *D. virilis* and *D. pseudoobscura*, a single *Sgs5*/*5bis* gene was identified. A phylogeny of all *Sgs5* and *Sgs5bis* amino acid sequences (Fig. 2) revealed a clear separation in the gene sequences of the two groups, *Sgs5* and *Sgs5bis*. The *D. virilis* gene (annotated as uncharacterized protein Dvir\GJ24445) and the *D. pseudoobscura Sgs5/5bis* gene (annotated as uncharacterized protein Dpse\GA20459) were clustered with the *Sgs5bis* genes and they shared with most other *Sgs5bis* sequences a motif Gln-Ala-Thr in the signal peptide. This suggests that *D. virilis* and *D. pseudoobscura* possess an ortholog of *Sgs5bis*. The *D. virilis* and *D. pseudoobscura* lineages diverged first in our sample (Fig. 1), but it cannot be determined whether they have lost *Sgs5* or if the *Sgs5-Sgs5bis* gene duplication arose after their separation. In this case, the ancestral gene before the duplication was probably *Sgs5bis.*

### Gains and losses of Sgs3, Sgs7, and Sgs8 genes

The genes *Sgs3*, *Sgs7* and *Sgs8* form a tight cluster, 4.5 kb long, on the 3 L arm in *D. melanogaster* [31] and share sequence similarities [16] in their N-terminal and C-terminal parts, however *Sgs3* contains internal repeats whereas *Sgs7* and *Sgs8* do not (Sgs7 and Sgs8 are small proteins, about 75 amino acids in length). When the internal repeats of *Sgs3* are excluded, the amino acid identity amongst the three genes in *D. melanogaster* is 51.3% between Sgs3 and Sgs7, 48.7% between Sgs3 and Sgs8, and 46.7% between Sgs7 and Sgs8. Additionally *Sgs3*, *Sgs7* and *Sgs8* share a phase 1 intron position, interrupting the signal peptide sequence [16]. In the clade *D. yakuba* / *santomea* / *erecta*, *Sgs7* and *Sgs8* are inverted with respect to the *D. melanogaster* arrangement (Fig. 1). *Sgs7* is duplicated in *D. yakuba* (Dyak\GE20214 and Dyak\GE21218) and *D. santomea* (Fig. 1) with the two copies being inverted relative to each other and having only one, nonsynonymous, nucleotide difference. *Sgs8* lies between the two *Sgs7* copies, and has the same orientation as *Sgs3*. In species outside the *D. melanogaster* subgroup, all the *Sgs3*, *Sgs7* and *Sgs8* sequences also have the same intron, with slightly different positions depending on codon indels before the intron. Notably, *D. suzukii* is the only species in our study that has lost *Sgs3. D. suzukii* retained *Sgs8* and has undergone an amplification of *Sgs7*, containing three identical copies.

In *D. pseudoobscura*, *D. ficusphila*, *D. rhopaloa* (see Fig. 1), *Sgs7* and *Sgs8* could not be identified. However, a BLAST search using the *Sgs7* or *Sgs8* sequences of *D. melanogaster* as queries, returned several *Sgs3*-like genes (i.e. long proteins with internal repeats showing N-terminal and C-terminal parts similar to *Sgs3*). In those species with no *Sgs7*, no *Sgs8* and several *Sgs3*-like genes occupying the physical location of *Sgs7* and *Sgs8*, it is tempting to infer that the ancestral *Sgs7* and *Sgs8* have gained internal repeats. According to such a hypothesis, at least in some cases, the non-repeated parts of those Sgs3-like protein sequences are expected to cluster with Sgs7/8.

**Fig. 3:**
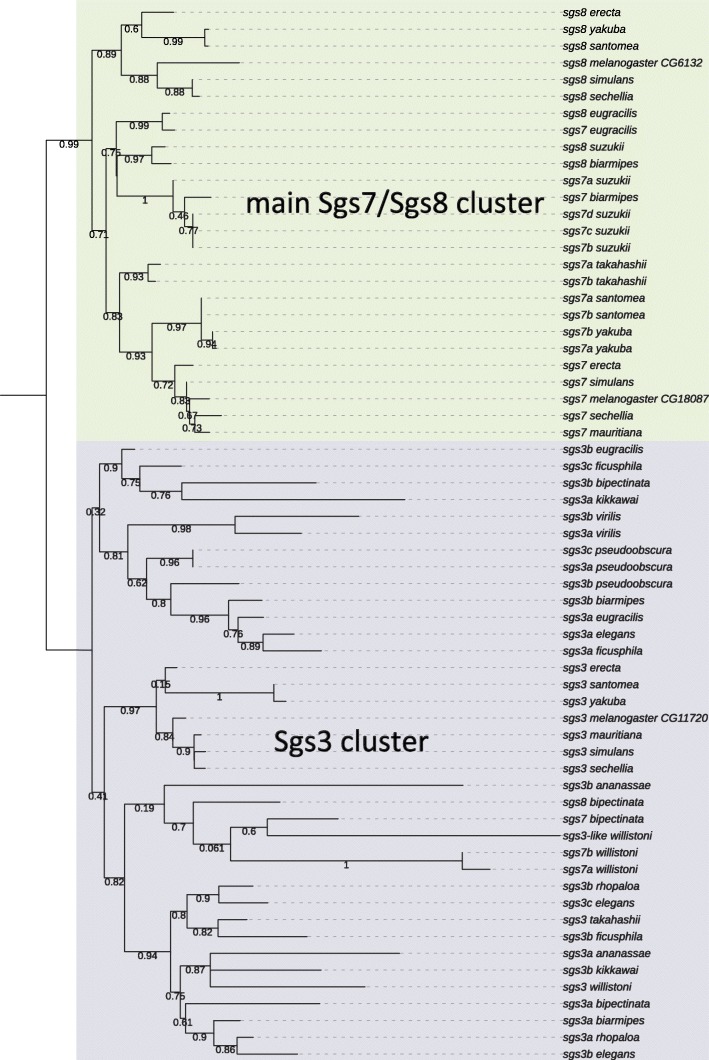
ML tree of aligned Sgs3 (repeats removed), Sgs7 and Sgs8 amino acid sequences. Numbers along branches are the posterior probabilities. The tree was rooted between the main Sgs7-Sgs8 cluster and the cluster containing all the Sgs3 sequences

To disentangle the relationships among *Sgs3*–*7-8* paralogs, we constructed a phylogeny using an alignment of the non-repeated parts of the protein sequences (Fig. 3). The tree is discordant with the assumed species phylogeny, but shows a clear separation between *Sgs3/Sgs3*-like and *Sgs7/Sgs8* genes. The exceptions are *D. bipectinata* and *D. willistoni*, whose *Sgs7/Sgs8* sequences are clustered with the *Sgs3* sequences, with low support due to short sequence lengths. This suggests that those *Sgs7/Sgs8* sequences are old *Sgs3*-like sequences which have lost their internal repeats. However, with such low support throughout the tree, we cannot confirm this hypothesis or infer whether there were two ancestral *Sgs3* and that subsequent losses occurred.

### Sgs1 is related to Sgs3/7/8

We find that *Sgs1* is only present in the *melanogaster* subgroup and so-called Oriental subgroups (with a loss in *D. erecta*), which suggests that it originated in the ancestor of this clade. The *Sgs1* sequence identified by BLAST search in the genome database (see Materials and Methods) was found to have many stop codons in the second half of the repeat region and had not been annotated as a coding sequence. However, based upon the surrounding repeat sequences, we found that inserting a C at position 1829 (from start) would restore the reading frame, translating into a putative 2245 amino acid protein. Inspection of another *D. suzukii* genome sequence [36] (contig CAKG01017146) showed that there is indeed a C at position 1829 and that Sgs1 is 2245 amino acid long, pointing to an error in the original annotation. Since position 1829 lies in the middle of a long repeat-containing region which prevents PCR amplification, we did not try to check experimentally for the missing C in the first *D. suzukii* genome sequence.

In all the *Sgs1* genes identified, except in *D. elegans*, an intron was found at the same position and phase as in *Sgs3*, *Sgs7* and *Sgs8*. There is also a loose similarity in the N-terminal and C-terminal parts of Sgs1 and Sgs3 (in *D. melanogaster* about 14% identity between Sgs3 and Sgs1 excluding the repeats). This suggests that *Sgs1* belongs to the same family as *Sgs3/Sgs7/Sgs8* genes*.*

### Origins of Sg4 and Eig71Ee genes remain elusive

*Sgs4* is intronless and is not present outside the *D. melanogaster* subgroup (Fig. 1, Table 2). We find no similarity between Sgs4 and any other sequence in any genome. Previously some sequence similarity between *Eig71Ee* and *Sgs4* had been reported [27], but only in the low complexity repeat regions of the genes. *Eig71Ee* is found in all the *D. melanogaster* subgroup species and in some of the so-called Oriental species, where it has been annotated as *mucin2*, or *extensin* in *D. takahashii*, or even, erroneously, as *Sgs3* in *D. suzukii*. We also detected the N-terminal parts of the gene in the *D. ananassae* group thus making the phylogenetic distribution of the gene unclear (Table 2). More interestingly, we noticed that *Eig71Ee* harbors an intron at the same position as the ones found in *Sgs3, Sgs7, Sgs8* and *Sgs1*. This result argues for a certain relatedness among those genes. However, using Eig71Ee as a TBLASTN query did not retrieve any hits from any *Sgs* genes and the Eig71Ee amino acid sequence does not align with the Sgs sequences.

### Rate of gene gains and losses in the glue gene families

Our analysis reveals that the seven annotated genes that code for glue proteins can be grouped into three gene families. *Sgs1, Sgs3, Sgs7, Sgs8,* and *Eig71Ee* comprise one of the three families since all of them share a phase 1 intron at the same position, interrupting the signal peptide sequence. *Sgs4* then forms its own family and the *Sgs5* and *5bis* comprise the third family. We used CAFE [37] to reconstruct ancestral copy numbers throughout the *Drosophila* phylogeny and to test whether these three gene families evolve at an accelerated rate along any *Drosophila* lineage. For the CAFE analysis *Eig71Ee* was not included due to uncertainties about its presence in some species. We find that the *Sgs4* and *Sgs5-5bis* families do not evolve faster compared to other gene families present in the *Drosophila* genomes (*p* = 0.58 and *p* = 0.107, respectively; Additional file 1: Table S1), however the *Sgs1–3–7-8* family was found to evolve rapidly (*p* = 0.005; Additional file 1: Table S1). Overall, this family seems to be prone to duplication and loss (Additional file 2: Figure S1) and we find that this signal for rapid evolution is driven mostly by small changes on many lineages (i.e. a gain or loss of 1 gene) rather than large changes on one or a few lineages.

### Characterization of the glue proteins and their repeats

Sgs1, Sgs3 and to a lesser extent, Sgs4 and Eig71Ee, are characterized by long repeats often rich in threonine and prone to O-glycosylations, in addition to their signal peptide and conserved C-terminus (Table 3 summarizes the characteristics of the repeats). We checked that across all populations in PopFly, the *D. melanogaster* Sgs5 protein is devoid of internal repeats while most other species, including close relatives of *D. melanogaster*, contain repeats mostly consisting in Pro-(Glu/Asp) pairs. Indeed the Sgs5 protein length is highly variable across species. For example, *D. kikkawai* harbors a long additional stretch (127 amino acids) containing 60% of acidic residues. Interestingly, the paralog Sgs5bis never has repeats. Sgs7 and Sgs8 are much smaller proteins, without any repeats and are rich in cysteine (12–14%). The conserved C-terminal sequences of Sgs proteins are important to characterize because the repeats are quite variable in motif, length and number, even between closely related species, meaning that, most often, glue proteins may be retrieved only based on their conserved C-terminal part. The C-terminal segments are about 120 amino acids long in Sgs1, 50 amino acids in Sgs3, 120 amino acids in Sgs4, 115 amino acids in Sgs5/5bis and 135 amino acids in Eig71Ee. The longest Sgs protein is Sgs1 in *D. suzukii* (2245 aa), which harbors ca. 63 repeats of a 29 amino acid, threonine-rich motif so that threonine makes up 40% of the residues. In *D. melanogaster*, Sgs1 is also very long (1286 aa) due to 86 repeats of a motif of 10 amino acids, also threonine-rich (46%). The shortest Sgs1 protein is the one of *D. sechellia* (492 aa). In all the species where it exists, Sgs1 is also rich in proline (12–18%). Sgs3 has a similar amino acid composition as Sgs1.

**Table 3:** Characteristics of glue proteins in the species studied (except Sgs7 and Sgs8)

Protein	Species	Length (aa)	Kind of repeat	Approx. nr of repeats	N glyc	O glyc	Disoredered repeats
Sgs1	*melanogaster*	1286	PTTTTPR/STTTTSTSR	ca 85	2	> 25	yes
	*simulans*	785	CAPTTTTPR	ca 40	1	> 25	yes
	*mauritiana*	412	CAPTTTTPR	ca 13	1	> 25	yes
	*sechellia*	492	CAPTTTTPR	ca 22	1	> 25	yes
	*santomea*		uncertain sequence				
	*yakuba*	619?	RPPTTSPSC	uncertain		> 25	
	*elegans*	837	T rich stretches		0	> 25	yes
	*rhopaloa*	ca. 624	T rich stretches		1	> 25	yes
	*ficusphila*	758	CAPTTTPST	ca 59	0	> 25	yes
	*takahashii*	585	TSTTTTPR	ca 25	1	> 25	yes
	*eugracilis*	635	PRCTTTTT	ca 39	0	> 25	yes
	*biarmipes*	696	VPTT/KCQMTTSSSAPTTAAPTATSTTAATTSTP	3/ca 12	1	> 25	yes
	*suzukii*	2245	VPTT/RCPITTSTSAPTTTTATTTSTSTSTTSTP	8/ca 63	1	> 25	yes
Sgs3	*melanogaster*	307	KPTTT	ca 31	0	> 25	yes
	*simulans*	188	a few T rich stretches		0	> 25	yes
	*mauritiana*	183	CAPPTRPPCTSPTTTTTTTTTT	ca 5	1	> 25	yes
	*sechellia*	172	CKPTTTTTT	ca 8	0	> 25	yes
	*santomea*	273	PTTTTTTTRR	ca 6	0	> 25	yes
	*yakuba*	273	PTTTTTTTRR	ca 6	0	> 25	yes
	*erecta*	333	TTRR	ca 35	3	> 25	yes
	*elegans a*	216	CAPTTTTTTTQR	ca 7	0	> 25	yes
	*elegans b*	202	KATT	ca 24	0	> 25	yes
	*elegans c*	287	PTTTTTKK	ca 23	1	> 25	yes
	*ficusphila a*	266	CAPTTTTTT	ca 12	0	> 25	yes
	*ficusphila b*	259	T rich stretches		0	> 25	yes
	*ficusphila c*	335	CKPPTTS/KPSKPT	ca 10/ca 28	1	> 25	yes
	*takahashii*	585	PTTTSTTR	ca 27	1	> 25	yes
	*eugracilis a*	214	CAPTTTTTTTTT	ca 7	0	> 25	yes
	*eugracilis b*	348	PTK	ca 65	2	> 25	yes
	*biarmipes a*	244	KKPXTT	ca 21	0	> 25	yes
	*biarmipes b*	302	T rich stretches		0	> 25	yes
	*rhopaloa a*	254	ATTK	ca 21	0	> 25	yes
	*rhopaloa b*	256	T rich stretches		0	> 25	yes
	*rhopaloa c*	253	CAPTTTTTT	ca 12	0	> 25	yes
	*rhopaloa d*	incomplete 5’	CAPTTTTTT	ca 9	0	> 25	yes
	*kikkawai a*	129	KPQP	ca 10	0	2	yes
	*kikkawai b*	190	KPQPP	ca 16	0	6	yes
	*ananassae a*	579	KPTTP	ca 55	1	> 25	yes
	*ananassae b*	566	PTR/PTE/PTV	ca 71/42/22	2	> 25	yes
	*bipectinata a*	272	T rich stretches/PTKSTR	ca 8	0	> 25	yes
	*bipectinata b*	254	QPPTKSTPKPT	ca 8	0	> 25	yes
	*pseudoobscura a*	207	KPT	ca 23	0	> 25	yes
	*pseudoobscura b*	229	KPTTTP	ca 14	0	> 25	yes
	*pseudoobscura c*	224	KPT	ca 33	0	> 25	yes
	*willistoni*	283	P/T-rich stretch		0	> 25	yes
	*willistoni sgs3-like*	546	CVTTRSSTPTP/CGPTPSPSPT	ca. 15/17	0	> 25	yes
	*virilis a*	242	RTTTTPTTTT	ca 12	0	> 25	yes
	*virilis b*	283	KPTTTRRT/KTIPTTTP	ca 11/9	2	> 25	yes
Sgs4	*melanogaster*	287	CRTEPPT	ca 19	0	> 25	yes*
	*simulans*	266	CDTEPPT	ca 8	0	> 25	yes*
	*mauritiana*	360	CNTEPPT	ca 31	0	> 25	yes*
	*sechellia*	255	CNTEPPT/CDTEPPT	ca5/4	0	> 25	yes*
	*santomea*	351	C(K/R)T(E/T)PPT / CKTKPPCTTV	ca 14/9	0	> 25	yes*
	*yakuba*	361	C(K/R)T(E/T)PPT	ca 23	0	> 25	yes*
	*erecta*	280	CRTEPPT/NAPTRRT	ca 8/7	1	> 25	yes*
Sgs5 and 5bis	*melanogaster*	163	no repeats		0	2	NA
	*melanogaster bis*	142	no repeats		0	0	NA
	*simulans*	169	PE/TE	ca 6	0	8	yes
	*simulans bis*	142	no repeats		0	0	NA
	*mauritiana*	169	PE/TE	ca 6	0	10	yes
	*sechellia*	169	PE/TE	ca 6	0	10	yes
	*sechellia bis*	142	no repeats		0	0	NA
	*santomea*	192	TE	ca 7	0	8	yes
	*santomea bis*	142	no repeats		0	0	NA
	*yakuba*	192	TE	ca 7	0	12	yes
	*erecta bis*	142	no repeats		0	0	NA
	*ficusphila*	208	DP or EP, ES, ET	ca 28	0	22	yes
	*ficusphila bis*	142	no repeats		0	0	NA
	*takahashii*	217	EP or EE	ca 12	0	19	yes
	*takahashii bis*	161	no repeats		0	3	NA
	*biarmipes*	190	PED or PET	ca 10	0	17	yes
	*biarmipes bis*	143	no repeats		0	1	NA
	*elegans*	223	EP	ca 27	0	11	yes
	*eugracilis*	187	PE	ca 16	0	14	yes
	*eugracilis bis*	142	no repeats		0	0	NA
	*suzukii*	203	PETE	ca 11	0	23	yes
	*suzukii bis*	142?	no repeats		0	1	NA
	*kikkawai*	362	PEDEED	ca 37	0	11	yes
	*kikkawai bis*	146	no repeats		0	2	NA
	*rhopaloa*	236	EP	ca 38	0	9	yes
	*ananassae*	172	almost no repeats		0	2	NA
	*ananassae bis*	146	no repeats		0	0	NA
	*bipectinata*	162	almost no repeats		0	3	NA
	*bipectinata bis*	146	no repeats		0	1	NA
	*pseudoobscura bis*	144	no repeats		0	0	NA
	*virilis*	143	no repeats		0	0	NA
Eig71Ee	*melanogaster*	445	CTCTESTT/(R/K)TNPT	ca 9/ca 7	8	> 25	yes
	*simulans*	321	CTCTDSTT(R/K)KTNPT	ca 4/ca 2	2	> 25	yes
	*sechellia*	408	CTDSTTKTTNPPCT	ca 8	3	> 25	yes
	*mauritiana*	284	no clear repeats		0	> 25	yes
	*yakuba*	417	CTESTTQKPNPPSTQKTRPPCG	ca 5	1	> 25	yes
	*santomea*	394	CTESTTQKPNPPSTEKTRPPCG	ca 3	1	> 25	yes
	*erecta*	454	CTESTTRRTKPPSTRKTRPP	ca 5	0	> 25	yes
	*ficusphila*	384	TE(K/R)T	ca 11	1	> 25	yes
	*takahashii*	302	CTEKTTQKPEPP	ca 7	0	> 25	yes
	*biarmipes*	434	no clear repeats		6	> 25	yes
	*suzukii*	346	no clear repeats		0	> 25	yes
	*eugracilis*	447	CTETTTQKTNPP	ca 5	0	> 25	yes

Glycosylation sites were predicted from http://www.cbs.dtu.dk/services/NetNGlyc/ and http://www.cbs.dtu.dk/services/NetOGlyc/ for N glycosylation and O glycosylation, respectively. *: except for IUPred and PrDOS

Repeats can also be quite different between paralogs. For example, in *D. eugracilis*, while the two *Sgs3*-like genes are physically neighbors, Sgs3a has several repeats of CAP(T)_n_, whereas Sgs3b has ca. 65 KPT repeats. In *D. elegans*, the three Sgs3-like proteins also have quite different repeats (Table 3). Sgs4 is richer in proline than in threonine (18% vs. 16% in *D. melanogaster*) and contains 10% cysteine residues.

### Interspecific variation in number and sequence of repeats

Between closely related species the number of repeats varied enormously and the repeated sequence diverged sometimes rapidly (Table 3). In the following we examine some specific examples to highlight these patterns. *D. simulans*, *D. sechellia,* and *D. mauritiana* form a clade, which split less than 300,000 years ago [38]. Their *Sgs1* genes harbor the same repeated sequence but the number of repeats ranges from 40 in *D. simulans* to 13 and 22 in *D. mauritiana* and *D. sechellia*, respectively. Likewise, Sgs3 is very similar in the three species, except in the number of repeats. There are no repeats in *D. simulans,* but threonine-rich stretches; in the published sequence of *D. mauritiana*, there are three tandem occurrences of CAPPTRPPCTSP(T)_n_; in *D. sechellia*, several CKP(T)_6_ repeats. Sgs4 shows shared repeats C(D/N)TEPPT among these species, with many more repeats in *D. mauritiana*. In contrast, in the sibling species *D. yakuba* and *D. santomea*, which diverged 0.5 million years ago [39, 40], *Sgs3*, *Sgs4* and *Sgs5* harbor the same repeat sequences and the same number of repeats (Table 3). *Sgs4* genes show 91% identity at the protein level with the same 23 repeats; Sgs5 97% identity and no repeats.

Another pair of species worth of interest is *D. suzukii*/*D. biarmipes*, considered to have diverged ca. 7.3 mya [41]. As mentioned above, only Sgs1 and Sgs5 can be compared because *D. suzukii* has lost *Sgs3*, and *Sgs4* is limited to the *melanogaster* subgroup. Despite a longer divergence time than for the previous comparisons, the Sgs1 29 amino acid repeats are similar in the two species but *D. suzukii* has many more repeat units. In the non repeat parts, identity is 69.3%; Sgs5 is well conserved even in the repeat region, with an overall identity of 76.4% in amino acids, and 84.8% in the non-repeat parts. A last pair of related species (despite their belonging to different subgroups) is *D. elegans/D. rhopalo*a. We estimate their divergence time to be roughly 12 million years based on molecular data (see Methods) and find that their Sgs proteins are very similar overall. This similarity extends to the repeat regions, with the exception being the repeats in Sgs3, which exists as four gene copies in *D. rhopalo*a. Their Sgs5 proteins have a high overall identity (75%), including repeats (Glu-Pro)_n_. In the non-repeat regions, identity rose to 82%. Indeed we often found more divergence among paralogs within a genome than across orthologous proteins.

**Fig. 4:**
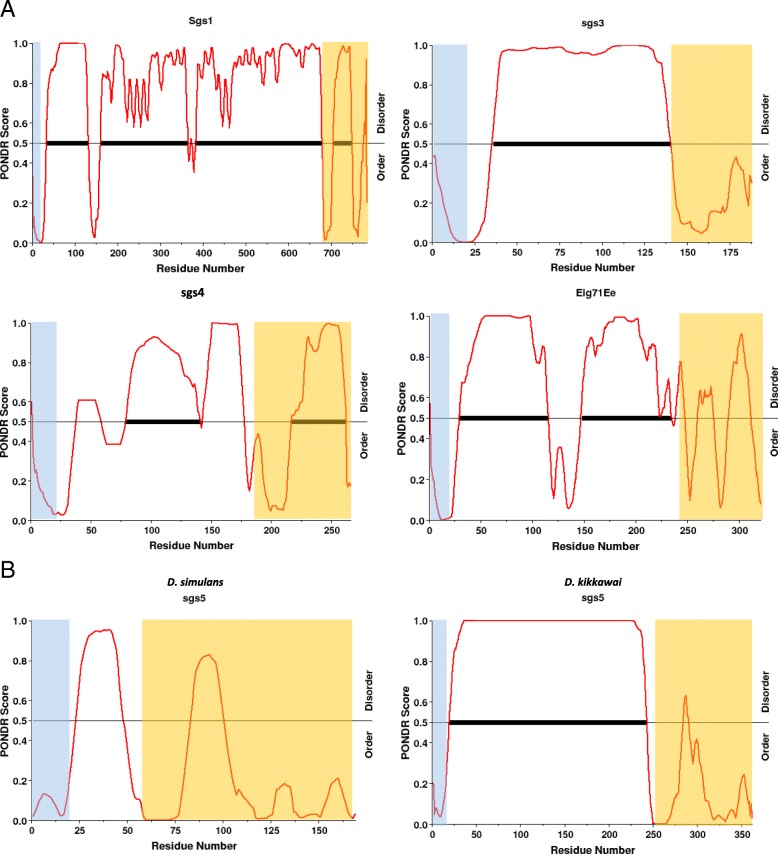
Example of predictions for disordered regions by PONDR. The X axis represents the protein length; the Y axis shows the score of the predictor VL-XT, which compares two predictors based on neural networks. The higher the value (closer to 1), the most disordered it is. The thick bars show the best predicted disordered regions. The VL-TX algorithm is more accurate for stretches longer than 30 amino acids. Regions shaded in light blue are the signal peptide regions; regions shaded in light orange are the conserved C-terminal regions. **a**: The glue proteins with internal repeats of *D. simulans,* except Sgs5; **b**: example of an Sgs5 protein with large internal repeats (*D. kikkawai*) compared to the one of *D. simulans*

Structure prediction programs (IUPred [42], PrDOS [43], disEMBL [44], PONDR [45]) indicate that the repeat regions of Sgs1, Sgs3, Sgs4, Sgs5 and Eig71Ee are intrinsically disordered (Fig. 4). Only IUPred and PrDOS indicate Sgs4 repeats to be ordered, in disagreement with the other predictors.

### Intraspecific variation in number of repeats

Owing to the difficulty of short-read sequencing methods to deal with the repeated sequences found in glue genes, we could not get a species-wide insight of repeat number variation (RNV) in *D. melanogaster*. Therefore, we resequenced *Sgs3* and *Sgs4* in strains from various geographic locations using classical Sanger sequencing (Table 4). We find striking inter- and intrapopulation variation in the number of repeats: for *Sgs3* (Additional file 3: Figure S2 and Additional file 4: Figure S3, Table 4), there was at least 9 repeat difference between the shortest and the longest allele (22 to 31); for *Sgs4*, we find a range of 18 to more than 26 repeats (Additional file 5: Figure S4 and Additional file 6: Figure S5, Table 4). Regarding the data from the *Drosophila* Genome Nexus study (Cairo population), we observed that the repeat region of *Sgs4* was erroneously reconstituted, often underestimating the repeat number, compared to our Sanger sequencing. We also sequenced the *Sgs3* and *Sgs4* genes in wild-caught *D. mauritiana* individuals. For *Sgs3* we found variation in the number of stretched threonines (10 or 12) and in the number of repeats (Additional file 7: Figure S6A and Table 4). For *Sgs4*, we found that the actual sequences were much longer than the sequence available online, and variable in length, even at the intra-population level, ranging from 25 to 35 repeats of the 7 amino acid motif (Additional file 7: Figure S6B and Table 4).

**Table 4:** List of strains used for PCR amplification. Number of repeats and repeat motifs in Sgs3 and Sgs4 in populations of *D. melanogaster* and *D. mauritiana*. Sequences of Sgs4 for Oregon R and Samarkand strains are from [83]. * indicate lines also used in the *Drosophila* Nexus project. @ indicate suspected artifactual repeat losses during cloning. PTC indicates the presence of a premature termination codon

protein	species	sample	Origin	nr of repeats	type of repeat	remarks
Sgs3	*D. melanogaster*	Cayenne	French Guyana	29	(K/N)(P/Q/A)TTT	
		Chavroche	France	29	(K/N)(P/Q/A)TTT	
		Chavroche2	France	29	(K/N)(P/Q/A)TTT	
		Chavroche3	France	30	(K/N)(P/Q/A)TTT	
		Cotonou	Benin	31	(K/N)(P/Q/A)TTT	
		Delhi1	India	27	(K/N)(P/Q/A)TTT	
		Delhi2	India	29	(K/N)(P/Q/A)TTT	
		Delhi B	India	27	(K/N)(P/Q/A)TTT	
		Gally A	France	29	(K/N)(P/Q/A)TTT	
		Gally B	France	29	(K/N)(P/Q/A)TTT	
		Gally C	France	29	(K/N)(P/Q/A)TTT	
		Gally D	France	29	(K/N)(P/Q/A)TTT	
		EF1 B	Ethiopia*	24	(K/N)(P/Q/A)TTT	
		EF1 3	Ethiopia*	29	(K/N)(P/Q/A)TTT	
		EG15N	Cairo, Egypt*	30	(K/N)(P/Q/A)TTT	
		EG16N	Cairo, Egypt*	> 25	(K/N)(P/Q/A)TTT	
		EG25N	Cairo, Egypt*	29	(K/N)(P/Q/A)TTT	
		EG28N	Cairo, Egypt*	> 29	(K/N)(P/Q/A)TTT	
		EG33N a	Cairo, Egypt*	12@	(K/N)(P/Q/A)TTT	
		EG33N c	Cairo, Egypt*	31	(K/N)(P/Q/A)TTT	
		EG34N	Cairo, Egypt*	7@	(K/N)(P/Q/A)TTT	
		EG55N	Cairo, Egypt*	23	(K/N)(P/Q/A)TTT	
		EG59N	Cairo, Egypt*	22	(K/N)(P/Q/A)TTT	
		EG74N	Cairo, Egypt*	23	(K/N)(P/Q/A)TTT	
	*D. mauritiana*	GM21	Grande Montagne (Rodrigues Island)	5	CAPPTRPP(T)n	
		GM23a	Grande Montagne (Rodrigues Island)	5	CAPPTRPP(T)n	
		GM23b	Grande Montagne (Rodrigues Island)	3	CAPPTRPP(T)n	
		GM24	Grande Montagne (Rodrigues Island)	4	CAPPTRPP(T)n	
		GM25	Grande Montagne (Rodrigues Island)	5	CAPPTRPP(T)n	
		GRNM1	Gorges de la Rivière Noire (Mauritius)	5	CAPPTRPP(T)n	
		MaurII-704	Mauritius	5	CAPPTRPP(T)n	
		MaurII-a	Mauritius	5	CAPPTRPP(T)n	
Sgs4	*D. melanogaster*	CG12181	reference strain Iso1	20	C(K/R/E)TEPP(R/T)	
		OregonR	lab strain (from [83])	22	C(K/R/E)TEPP(R/T)	
		Samarkand	[83]	21	C(K/R/E)TEPP(R/T)	
		Canton S	Lab strain	> 21	C(K/R/E)TEPP(R/T)	
		Cayenne1	French Guyana	> 21	C(K/R/E)TEPP(R/T)	
		Cayenne2	French Guyana	> 22	C(K/R/E)TEPP(R/T)	
		Cayenne3	French Guyana	> 21	C(K/R/E)TEPP(R/T)	
		Chavroche1	France	> 22	C(K/R/E)TEPP(R/T)	
		Chavroche3	France	> 22	C(K/R/E)TEPP(R/T)	
		Comores1	Comores	> 22	C(K/R/E)TEPP(R/T)	
		Comores2	Comores	> 22	C(K/R/E)TEPP(R/T)	
		Cotonou	Benin	> 22	C(K/R/E)TEPP(R/T)	
		Delhi1	India	> 21	C(K/R/E)TEPP(R/T)	
		Delhi2	India	> 21	C(K/R/E)TEPP(R/T)	
		Gally1	France	> 20	C(K/R/E)TEPP(R/T)	
		Gally2	France	> 20	C(K/R/E)TEPP(R/T)	
		EF1	Ethiopia*	> 22	C(K/R/E)TEPP(R/T)	
		Tai1	Ivory Coast	> 20	C(K/R/E)TEPP(R/T)	
		Tai2	Ivory Coast	> 20	C(K/R/E)TEPP(R/T)	
		EG15N	Cairo, Egypt*	> 26	C(K/R/E)TEPP(R/T)	PTC
		EG16N	Cairo, Egypt*	22	C(K/R/E)TEPP(R/T)	PTC
		EG25N	Cairo, Egypt*	20	C(K/R/E)TEPP(R/T)	PTC
		EG28N	Cairo, Egypt*	20	C(K/R/E)TEPP(R/T)	PTC
		EG33N	Cairo, Egypt*	20	C(K/R/E)TEPP(R/T)	PTC
		EG34N	Cairo, Egypt*	22	C(K/R/E)TEPP(R/T)	PTC
		EG36N	Cairo, Egypt*	22	C(K/R/E)TEPP(R/T)	PTC
		EG44N	Cairo, Egypt*	> 26	C(K/R/E)TEPP(R/T)	PTC
		EG55N	Cairo, Egypt*	> 26	C(K/R/E)TEPP(R/T)	PTC
		EG59N	Cairo, Egypt*	> 26	C(K/R/E)TEPP(R/T)	PTC
		EG74N	Cairo, Egypt*	> 26	C(K/R/E)TEPP(R/T)	
		ZI395	Zambia*	25	C(K/R/E)TEPP(R/T)	
		ZI420	Zambia*	18	C(K/R/E)TEPP(R/T)	
	*D.mauritiana*	GM22	Grande Montagne (Rodrigues Island)	> 30	C(N/D)TEPP	
		GM23	Grande Montagne (Rodrigues Island)	> 31	C(N/D)TEPP	
		GM25	Grande Montagne (Rodrigues Island)	> 30	C(N/D)TEPP	
		GRNM1	Gorges de la Rivière Noire (Mauritius)	> 27	C(N/D)TEPP	
		GRNM2	Gorges de la Rivière Noire (Mauritius)	> 32	C(N/D)TEPP	
		GRNM3	Gorges de la Rivière Noire (Mauritius)	> 27	C(N/D)TEPP	
		GRNM6	Gorges de la Rivière Noire (Mauritius)	> 24	C(N/D)TEPP	
		MaurII-a	Mauritius	> 28	C(N/D)TEPP	
		MaurII-704	Mauritius	> 28	C(N/D)TEPP	
Sequence checking	*D. sechellia*		Praslin Island			
	*D. santomea*	STO3	Sao Tomé			
	*D. virilis*		Spain			
	*D. biarmipes*		India			

### Nonsense mutations in the Sgs genes

Despite the rather low quality of sequences in the *Drosophila* Genome Nexus data set, we searched for putative premature termination codons (PTC) in *Sgs* genes of *D. melanogaster*, which could lead to non-functional proteins. The search was limited to non-repeat regions. We find PTC in *Sgs4* of several lines that truncated the protein at the beginning of its conserved C-terminal part. We confirmed experimentally the presence of this PTC in 10 lines of the Cairo population EG (K165stop) (Additional file 6: Figure S5 and Table 4). We also found putative PTC for *Sgs5* in a few lines (W161stop, that is sub-terminal, and maybe not detrimental), and experimental verification confirmed it in one Ethiopian line (EF66N); in *Sgs5bis*, we found a putative PTC (C33stop) in six African lines from Rwanda (RG population) and Uganda (UG population). We also find a putative PTC for *Sgs1* in a few lines from USA and Cairo (P49stop), which was confirmed by resequencing the Egyptian line EG36N. This nonsense mutation required two substitutions from CCA to TAA in all cases. Interestingly, EG36N also has a truncated Sgs4, warranting more careful investigation of its glue gene.

In *Sgs3*, no PTC was found, but putative PTC were found for Eig71Ee in two lines, EA90N (S345stop) and RAL894 (W380stop), both in the C-terminal region. One putative PTC was found in *Sgs7* (Q47stop, line USI33), but was not checked experimentally. No PTC was found in *Sgs8* sequences. Stretches of Ns found in non-repeat regions could possibly, at least in some cases, turn out to be true deletions, which deserves further investigation. There is a possibility that some PTCs could experience stop codon readthrough [46] leading to translation of the correct protein. For instance this is possible in Sgs4 because the nonsense mutation was not accompanied by other mutations, which would be expected in case of relaxed selection (unless the nonsense mutation is very recent). Further studies of the protein content of the salivary glands in those strains will be needed to check whether Sgs4 is produced and if it is full-size.

### Evolutionary rate of Sgs protein sequences

**Fig. 5:**
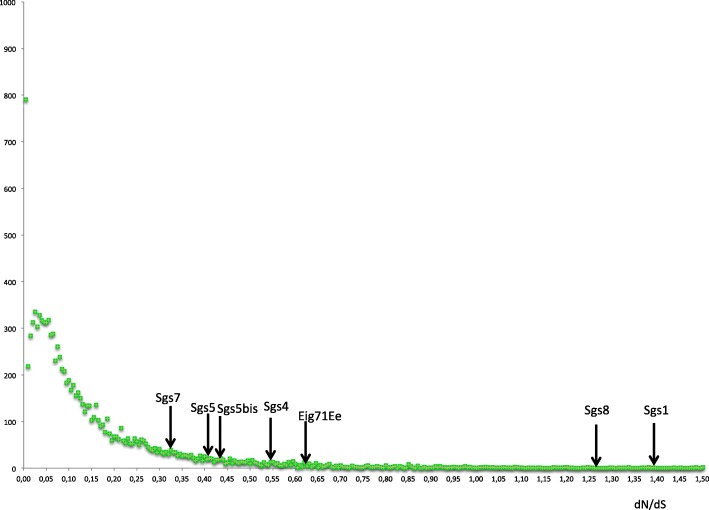
Distribution of dN/dS for the pair *D. melanogaster*/*D. simulans* from the flyDIVaS database with the position of glue genes. Vertical axis: number of genes. Genes are binned into rate value categories with increment of 0.005

Given that glue proteins harbor RNV and our hypothesis that they could be putative targets for fast selection, we wanted to test whether glue gene coding sequences evolve quickly. To this end, we computed substitution rates of the *Sgs* genes between *D. melanogaster* and *D. simulans* (Table 5). We did not include *Sgs3*, because the internal repeats were very different and not alignable between the two species. This, at any rate, shows that this particular gene has evolved rapidly. Although it had the biggest size and the highest number of repeats, we were able to make an estimate for *Sgs1* because the repeats were rather similar in *D. melanogaster* and *D. simulans.* We removed the unalignable parts before computation, therefore underestimating the real evolutionary rate. Rate calculations were similarly performed for *Eig71Ee*, *Sgs4* and *Sgs5*. The computed values for the Sgs genes were compared to the genome-wide distributions of dN/dS between these species (Fig. 5) using data from the flyDIVaS database [47]. All dN values were within the highest quartile, and *Sgs1*, *Sgs4* and *Sgs8* were within the highest three centiles. Furthermore, high dN/dS values were found for *Sgs1* (dN/dS = 1.393) and *Sgs8* (dN/dS = 1.259), indicating accelerated protein evolution. The dN value of *Sgs8* (0.1789) contrasts with the one of its close relative *Sgs7* (0.0475).

**Table 5:** Non-synonymous (dN) and synonymous (dS) substitution rates, and the dN/dS ratio for glue genes between *D. melanogaster* and *D. simulans* in pairwise alignments. *Sgs3* was not included, and unalignable regions were removed

	*Sgs1*	*Sgs4*	*Sgs5*	*Sgs5bis*	*Sgs7*	*Sgs8*	*Eig71Ee*
dN	0.110	0.183	0.034	0.029	0.047	0.179	0.0678
dS	0.079	0.334	0.084	0.067	0.146	0.146	0.110
dN/dS	1.393	0.547	0.405	0.430	0.323	1.259	0.616

We wondered if *Sgs8* had also evolved faster than *Sgs7* in other pairs of related species. Table 6 shows the results for other species pairs known to be close relatives: *D. melanogaster/D. sechellia*, *D. simulans/D. sechellia*; *D. yakuba/D. erecta*; *D. biarmipes/D. suzukii*. Comparisons between *D. yakuba/D. erecta* and *D. biarmipes/D. suzukii* showed no evolutionary rate difference between *Sgs7* and *Sgs8*. However we found that between *D. simulans* and *D. sechellia Sgs7* has a dN ten times higher than that of *Sgs8*. This pattern is opposite that the *D. simulans* vs. *D. melanogaster* comparison. In fact, *D. sechellia Sgs7* is more divergent than *D. simulans* from *D. melanogaster Sgs7*, whereas *Sgs8* has not diverged further. Obviously, the small number of substitutions points to a high variance, and the difference may be not significant.

**Table 6:** Non-synonymous (dN) and synonymous (dS) substitution rates and the ratio dN/dS for *Sgs7* and *Sgs8* between related species pairs in pairwise alignments

Species pair	Gene	dN	dS	dN/dS
*melanogaster/simulans*	*Sgs7*	0.0475	0.1459	0.323
	*Sgs8*	0.1789	0.1420	1.259
*melanogaster/sechellia*	*Sgs7*	0.0990	0.1339	0.739
	*Sgs8*	0.1866	0.1216	1.534
*simulans/sechellia*	*Sgs7*	0.0696	0.0559	1.245
	*Sgs8*	0.0060	0.0564	0.106
*yakuba/erecta*	*Sgs7*	0.1780	0.2235	0.796
	*Sgs8*	0.1623	0.2164	0.750
*biarmipes/suzukii*	*Sgs7*	0.0592	0.4329	0.137
	*Sgs8*	0.0565	0.4533	0.125

To test for adaptive evolution after the “out of Africa” event of *D. melanogaster* [48], we measured the nucleotide diversity π and divergence D_xy_ between one population from Zambia, (ZI) thought to be within the original geographical area of *D. melanogaster*, another African population (EF, Ethiopia) and two derived populations, from France (FR) and USA (Raleigh, RAL). This study was limited to the coding sequences of *Sgs5* and *Sgs5bis* because these genes conveniently lack internal repeats and the gene size is not too short, (as opposed to *Sgs7* and *Sgs8*)*.* Due to the numerous residual unidentified nucleotides in the *Drosophila* Genome Nexus data, the number of sites taken into account was actually much smaller than the sequence size, e.g. for *Sgs5bis*, 278 sites left over 489 in RAL. We compared the overall π and D_xy_ between these populations [49]. Roughly, for both genes π is higher in ZI than in EF, FR and RAL (Tables 7 and 8). This matches the pattern observed for the whole genome and is as expected for the region of origin of this species. We found that divergences D_xy_ are less than expected from the whole genome, except for the ZI/EF comparison of *Sgs5* (Tables 7 and 8). Both genes gave similar results. Therefore, we find that the glue genes *Sgs5* and *Sgs5bis* do not show particular divergence pattern across populations, which could have been related to a change in population environment.

**Table 7:** Nucleotide diversity π of *Sgs5* and *Sgs5bis* in four populations, computed from Jukes and Cantor [84] using DnaSP

*Sgs5*	N	n	S	π (S.D.)	π_global_
EF	35	467	11	0.00450 (0.00106)	0.00622
FR	45	476	5	0.00423 (0.00023)	0.00471
ZI	183	489	38	0.00998 (0.00030)	0.00843
RAL	153	386	8	0.00257 (0.00015)	0.00569
*Sgs5bis*	N	n	S	π (S.E.)	π_global_
EF	35	406	3	0.00267 (0.00024)	0.00622
FR	45	422	8	0.00460 (0.00029)	0.00471
ZI	201	426	37	0.00614 (0.00034)	0.00843
RAL	172	278	5	0.00322 (0.00018)	0.00569

**Table 8:** Nucleotide divergence between populations D_xy_ computed from Jukes and Cantor [84]in DnaSP

*Sgs5*	N	n	S	D_xy_ (S.D.)	D_□□ global_
ZI/EF	183/35	467	37/11	0.01197 (0.00082)	0.00855
ZI/FR	183/45	476	33/5	0.00685 (0.00046)	0.00868
ZI/RAL	183/153	386	25/8	0.00488 (0.00036)	0.00864
EF/FR	35/45	454	8/5	0.00810 (0.00128)	0.00795
EF/RAL	35/153	373	6/8	0.00705 (0.00093)	0.00790
FR/RAL	45/153	379	7/2	0.00162 (0.00025)	0.00546
*Sgs5bis*	N	n	S	D_□□_ (S.D.)	D_□□ global_
ZI/EF	201/35	406	35/3	0.00506 (0.00055)	0.00855
ZI/FR	201/45	422	36/8	0.00639 (0.00057)	0.00868
ZI/RAL	201/172	278	23/5	0.00423 (0.00033)	0.00864
EF/FR	35/45	402	3/6	0.00477 (0.00091)	0.00795
EF/RAL	35/172	263	3/5	0.00551 (0.00090)	0.00790
FR/RAL	45/172	276	6/5	0.00289 (0.00035)	0.00546

EF: Ethiopia, FR: France, ZI: Zambia, RAL: Raleigh. N: number of lines, n: number of sites, S: number of segregating sites, S.D.: standard deviation, π_global_ and D_xyglobal_: nucleotide diversity and nucleotide divergence across the genomes, respectively, from [49]

We also searched for episodic diversifying selection (EDS) among species for the three genes entirely devoid of repeats, *Sgs5bis*, *Sgs7* and *Sgs8* using the branch-site REL test (BS-REL) from the HyPhy package. No accelerated evolution was detected for *Sgs5bis*, whereas one branch (*D. santomea-D. yakuba* clade) underwent EDS for *Sgs7* (corrected *p*-value 0.012) and one branch (*D. erecta-D. yakuba-D. santomea)* underwent EDS for *Sgs8* (corrected p-value 0.015) (Additional file 8: Figure S7). These results must be considered with caution given the small size of the data set, but anyway do not favor a specific selection regime, regarding single nucleotide (or amino acid) polymorphism.

## Discussion and conclusion

We have investigated the presence and characteristics of *Sgs* genes and proteins in several *Drosophila* species belonging to the two main subgenera *Sophophora* and *Drosophila*, with particular emphasis on species closer to *D. melanogaster*. We have identified the various *Sgs* genes through sequence similarity with *D. melanogaster*. While this study is extensive, it is of course possible that we may have missed glue genes completely different from the ones of *D. melanogaster*. In order to get the full collection of glue genes we require transcriptional evidence from late larval salivary gland RNA for each species studied. Interestingly, according to our census, the seven genes characterized for years in *D. melanogaster* are far from being always present in the other genomes, although the seven members are generally preserved in the *D. melanogaster* subgroup. Our results are in disagreement with the succinct interspecific study of Farkaš [50]. We also propose here an eighth glue gene, *Sgs5bis.* Based on its close sequence homology and its co-expression with *Sgs5* we propose that these two genes are tandem paralogs. We notice that *Sgs5bis* never contains internal repeats whereas *Sgs5* often harbors more or less developed repeat motifs, although not in *D. melanogaster*. Given our data, and notwithstanding the unbalanced taxonomic sampling which may mislead us, we suggest that the ancestor of the species studied here had only *Sgs3* and *Sgs5bis* (Fig. 1). It is likely that *Sgs7*, *Sgs8*, and perhaps also *Sgs1* and *Eig71Ee*, originated from duplications of *Sgs3*. The important differences in repeat motifs between duplicate *Sgs3* (e.g. in *D. eugracilis*) are striking and suggest a high rate of evolution, or independent acquisition of repeats from a repeatless or repeat-poor parental gene. A part of the sequence we named *Sgs3-like* in *D. willistoni* is reported in FlyBase as GK28127, with transcription on the opposite strand, and without a homolog in *D. melanogaster*. Thus, it is possible that some duplicates of *Sgs3* may have been actually recruited for other functions other than glue production. In this respect, it is also possible that Eig71Ee, which has been studied mostly for its immune functions, could be an ancient glue protein, which gained new functions.

The repeat-containing glue proteins are typical of secreted mucins. Mucins are highly glycosylated proteins found in animal mucus and they protect epithelia from physical damage and pathogens [51]. In *D. melanogaster*, more than 30 mucin-like proteins have been identified [52] but the precise function of most of them remain unknown. It would be interesting to compare the glue genes with the other mucin-like genes in terms of protein domains and sequence evolution. In *D. melanogaster*, repeats similar to those of Sgs3 (KPTT) are found in the mucin gene *Muc12Ea*. The high level of glycosylation is thought to favor solubility at high concentration while accumulating in salivary glands ([50]). The richness in cysteines suggests that, upon release in the environment through expectoration, disulfide bridges between glue proteins may be formed by cysteine oxidation by air, making a complex fibrous matrix. Intramolecular disulfide bonds can also be predicted ([50]). Examination of the amino acid composition of the glue proteins suggests that the numerous prolines may induce a zigzag-like shape while serine and threonine, which are very abundant, besides being prone to O-glycosylation, make them very hydrophilic and favor interaction with the solvent and then solubility while preventing folding. The presence of regularly scattered arginines or lysines (or sometimes aspartic and glutamic acids) would add charge repulsion, helping the thread structure to be maintained flat and extended. This is similar to linkers found between mobile domains in some proteins [53]. The shorter Sgs7/Sgs8 would, considering their richness in cysteine, bind the threads together through disulfide bonding.

In the frame of an intrinsically disordered structure (Fig. 4), it is not surprising to observe a high level of repeat number variation (RNV) even at the intra-population level. It has been reported ([54, 55]) that in proteins with internal domain or motif repeats, if these repeats form disordered regions and do not interact with the rest of the protein chain (for a cooperative folding for example), they are more prone to indels which are better tolerated, and favored by the genetic instability of repeated sequences. It is likely that, within a certain repeat number range, variations in repeat numbers might have little effect on the chemical and mechanical properties of the glue. In fact it is likely that the differences in repeat motif sequences rather than the number of repeats would change the mechanical and physical properties of the glue. Accordingly, we measured rather fast rates of evolution, but found no clear indication of positive selection. One reason why the evolution of the repeats is fast (across related species or across paralogs) might be that the constraints to maintain disorder and the thread-like shape are rather loose ([54]).

We do not know the respective roles of the different Sgs proteins in the final glue. Farkaš [50] mentioned that Sgs1 could have chitin-binding properties, which is in line with the function of the glue. He also proposed roles of specific components before expectoration, inside salivary gland granules, related to packaging, solubility. The absence of some glue components may have consequences on its properties and may play a role in adaptation, as suggested by [50]. Gene loss, gene duplication, or repeat sequence change may modify the strength of the glue or its resistance to water or moisture, to acidity (of a fruit) and therefore might be linked to pupariation site preference. *D. suzukii* lacks both Sgs3 and Sgs4, and has duplications of Sgs7. *D. suzukii* pupae are found mostly in the soil just below the surface, and less rarely within ripe and wet fruits such as cherries or raspberries, the pupa half protruding [56, 57]. The extensive loss of *Sgs* genes in *D. suzukii* may be related to its pupariation in soil. Shivanna et al. ([58]) have related pupariation site preference to the quantity of glue and, counter-intuitively, have reported that species that prefer to pupariate on the food medium in the laboratory produce more glue than species that pupariate on the glass walls of the vials. However, the chemical glue content was not investigated. Another study [59] compared pupariation site preferences between the sibling species *D. mauritiana*, *D. sechellia* and *D. simulans*. While *D. simulans* populations from the native region share pupariation preference in fruits with *D. mauritiana* and *D. sechellia*, worldwide populations preferably pupariate off-fruit, i.e. on a drier and harder substrate. Although the QTL associated with pupariation site preference in *D. simulans* and *D. sechellia* do not map to glue genes [59], it would be interesting to see whether, secondarily, significant variations in glue composition or quantity occurred and might be contrasted across *D. simulans* populations. Given its worldwide expansion associated with adaptation to multiple local environments including diverse pupariation sites, *D. melanogaster* is an interesting model to study the intraspecific evolution of *Sgs* genes in relation to adaptation. Interestingly, absence of Sgs4 protein was reported in a few strains from Japan and USA [33], most likely due to deletions or mutations in the promoter region. Our resequencing of a few Nexus lines revealed nonsense mutations within the coding sequence at position 165 in *Sgs4*, deleting the well conserved C-terminal part*.* The translational consequences for this protein and for final glue properties remain unknown. In addition to such qualitative protein variations, it is possible that the relative proportions of the Sgs proteins in the glue may change in *D. melanogaster* according to the ecological circumstances. In this respect, collecting wandering larvae from various substrates, analyzing their glue composition and designing adhesion assays to compare adhesive properties between various glues will be valuable.

In conclusion, the pupal glue appears as a genetically and phenotypically simple model system for investigating the genetic basis of adaptation. The present work provides a first exploration of the evolution of glue genes across *Drosophila* species and paves the way for future studies on the functional and adaptive consequences of glue composition variation in relation to habitat and geographic and climatic origin.

## Methods

### Identification of Sgs genes in Drosophila species

The seven annotated glue genes of *D. melanogaster (Sgs1* (CG3047)*; Sgs3* (CG11720)*; Sgs4* (CG12181)*; Sgs5* (CG7596)*; Sgs7* (CG18087)*; Sgs8* (CG6132)) and *Eig71Ee* (CG7604) were used as BLAST queries for retrieving their orthologs in 19 other *Drosophila* species. The genome data used for each species is indicated in Table 1. BLAST searches were performed directly through GenBank, FlyBase [60], the SpottedWingFly base for *D. suzukii* [61] or using local BLAST program (v2.2.25) after downloading the genomes for *D. santomea* [62] and *D. mauritiana* [63]*.* The BLASTP and TBLASTN programs were used [64], without filtering for low complexity, which otherwise would have missed the repeated regions. Repeats, when present, were often quite different from the repeats present in *D. melanogaster Sgs* sequences. Consequently, BLAST results were often limited to the C-terminal part of the targeted gene, which was the most conserved part of the proteins, and to a lesser extent to the N-terminal end. For each species, a nucleotide sequence containing large regions upstream and downstream of the BLAST hits was downloaded from InsectBase [65] or from species-specific websites when genome data was not present in InsectBase (Table 1). We used Geneious (Biomatters Ltd.) to identify by eye the coding regions, the start of which was identified by the signal peptide sequence. Putative introns were also identified manually, guided by the intron-exon structure of the *D. melanogaster* orthologs. In cases of uncertainties or missing sequence data, we extracted DNA from single flies of the relevant species (Table 4) and the questionable gene regions were amplified with primers chosen in the reliable sequence parts (Additional file 9: Table S2), and sequenced by the Sanger method using an ABI 3130 sequencer. For instance, we characterized the exact sequence corresponding to N stretches in the published sequence of *D. mauritiana Sgs4*; we found that the published premature termination codon (PTC) of *D. biarmipes Sgs3* was an error and that three frameshifts found within 50 bp in *D. sechellia Sgs1* were erroneous.

### Evolutionary relationships between genes and estimate of evolutionary rates

Alignments of DNA or protein sequences were done using MUSCLE [66] implemented in Geneious and protein trees were computed using PhyML, as implemented in the online server Phylogeny.fr [67], drawn using iTOL [68], and rooted at midpoint. The substitution rates dN and dS values for over 10,000 coding sequences computed for *D. melanogaster/D. simulans* comparisons were retrieved from the flyDIVaS database [47] but *Sgs* genes were not included in this dataset. Thus, dN and dS were computed using yn00 in the PAML package ([69]), removing the unalignable parts. We tested for episodic diversifying selection across species using the branch-site random effect likelihood (BS-REL) algorithm implemented in the HyPhy package [70, 71] at the Datamonkey server (classic.datamonkey.org) [72]. We used only genes devoid of repeats to ensure reliable aligments, and we supplied species trees for the analysis.

### Test for accelerated gene turnover

To infer ancestral gene counts in the three newly classified *Sgs* gene families and to determine whether the three newly classified *Sgs* gene families are evolving rapidly we first need to determine the average rate of gene gain and loss (*λ*) throughout *Drosophila*. Previous studies have estimated *λ* from 12 *Drosophila* genomes and found rates of 0.0012 gain/losses per million years [4] and 0.006 gains/losses per million years after correcting for assembly and annotation errors [37]. However, since those studies numerous additional *Drosophila* genomes have been published. In order to update the gene gain/loss rate (*λ*) for this genus, we obtained 25 available *Drosophila* peptide gene annotations from NCBI and FlyBase. The latest versions at the time of study for the genomes of the original 12 sequenced species (*ananassae* v1.05*, erecta* v1.05*, grimshawi* v1.3*, melanogaster* v6.10*, mojavensis* v1.04*, persimilis* v1.3*, pseudoobscura* v3.04*, sechellia* v1.3*, simulans* v2.02*, virilis* v1.06*, willistoni* v1.05*i,* and *yakuba* v1.05) were downloaded from FlyBase [73] and 13 other species (*arizonae, biarmipes, bipectinata, busckii, elegans, eugracilis, ficusphila, kikkawai, miranda, navojoa, rhopaloa, suzukii,* and *takahashii*) were downloaded from NCBI [74].

To ensure that each gene from the 25 *Drosophila* species was counted only once in our gene family analysis, we used only the longest isoform of each protein in each species. We then performed an all-vs-all BLAST search [75] on these filtered sequences. The resulting e-values from the search were used as the main clustering criterion for the MCL (Markov cluster algorithm) program to group peptides into gene families [76].This resulted in 17,330 clusters. We then removed all clusters not present in the *Drosophila* ancestor, resulting in 9379 gene families. An ultrametric phylogeny with branch lengths in millions of years (my) was inferred using MCL in a similar fashion, with the addition of the genome of the house fly, *Musca domestica*, as an outgroup and utilizing single-copy orthogroups between all 26 species [77]. Calibration points at the split of *D. pseudoobscura*/*D. melanogaster* (49–59 my), *D. melanogaster*/*D. grimshawi* (64–74 my), and *Musca domestica*/*D. melanogaster* (156 my) were from Timetree.org [78, 79].

With the gene family data and ultrametric phylogeny as input, we estimated gene gain and loss rates (*λ*) with CAFE v3.0 [4]. This version of CAFE is able to estimate the amount of assembly and annotation error (*ε*) present in the input data using a distribution across the observed gene family counts and a pseudo-likelihood search. CAFE is then able to correct for this error and obtain a more accurate estimate of *λ*. We find an *ε* of about 0.04, which implies that 4% of gene families have observed counts that are not equal to their true counts. After correcting for this error rate, we find *λ* = 0.0034. This value for *ε* is on par with those previously reported for *Drosophila* (Additional file 10: Table S3; [37]). However, this *λ* estimate is much higher than the previous reported from 12 *Drosophila* species (Additional file 10: Table S3; [4, 37]), indicating a much higher rate of error distributed in such a way that CAFE was unable to correct for it, or a much higher rate of gene family evolution across Drosophila than previously estimated. The 25 species *Drosophila* phylogeny was then manually pruned and modified to represent the 20 *Drosophila* species in which *Sgs* gene families have been annotated. Some *Sgs* gene families are not present in the ancestor of all 20 species, so additional pruning was done to the phylogeny for each family as necessary (see Additional file 1: Table S1). The phylogeny, *Sgs* gene copy numbers, and the updated rate of gene gain/loss (*λ* = 0.0034) were then used by CAFE to infer *p*-values in each lineage of each family (Additional file 11: Table S4). Low p-values (< 0.01) may indicate a greater extent of gene family change along a lineage than is expected with the given *λ* value, and therefore may represent rapid evolution.

### *Search for polymorphism and repeat number variation in D. melanogaster and* D. mauritiana

Polymorphism in *D. melanogaster* was investigated in the coding regions, especially the repeat number variation (RNV). We intended to use the data from the *Drosophila* Genome Nexus study ([49, 80], available at the Popfly web site [81]) to assess RNV. This database contains resequenced and aligned genomes of hundreds of *D. melanogaster* lines from about 30 populations from all over the world. Those data, like most *D. melanogaster* populations’ and other species’ genomes were obtained using NGS technologies, which yielded short reads. The data were often not accurate in repeat regions, likely because short reads may be not properly assembled when there are numerous short tandem repeats, and thus could not be used for counting RNV. Thus, experimentally, using single-fly DNAs, we amplified and sequenced the repeat-containing *Sgs3* and *Sgs4* from one or a few individual flies from several strains or natural populations available at the laboratory (French Guyana, Ethiopia, France, Benin, Ivory Coast, India, Comores, and the laboratory strain Canton S), and from a number of lines used in the *Drosophila* Genome Nexus study (Table 4). In addition, we investigated the occurrence of possible premature termination codons in gene alignments from the *Drosophila* Nexus database [49, 80], available at the Popfly web site [81] and checked the results by PCR in *Sgs4* and *Sgs5* (Table 4). We also used data from the *Drosophila* Nexus database to study polymorphism and divergence in *Sgs5* and *Sgs5bis*, which are devoid of repeats, and are not too short. Four populations represented by numerous lines were retained for analysis: ZI (Siavonga, Zambia), for the ancestral geographical range, EF (Fiche, Ethiopia), which shows overall rather large differentiation (Fst) with most other populations [49], and FR (France) and RAL (Raleigh, USA) for the worldwide populations. Diversity and divergence indices were computed with DnaSP [82]. Experimental sequences were deposited to GenBank with accessions MH019984-MH020055.

## Additional files

Additional file 1: **Table S1.** Number of gene copies for each family, and results of CAFE analysis for the glue gene families. (XLSX 161 kb)

Additional file 2: **Figure S1.** Ancestral states for the *Sgs1–3–7-8* gene family inferred by CAFE. Species tips are labeled with the observed gene count and internal nodes are labeled with inferred gene counts. Orange branches represent gene losses, blue branches represent gene gains, while black branches represent lineages in which no change in gene copy number is observed. Branches marked with asterisks have marginally significant *p*-values (< 0.05). (PDF 173 kb)

Additional file 3: **Figure S2.** Partial alignment of *Sgs3* sequences with translation in *D. melanogaster* individuals. EF: Ethiopia; Chavroche and Gally: France; Cotonou: Benin; Delhi: India; Cayenne: French Guyana. (PDF 2258 kb)

Additional file 4: **Figure S3.** Partial alignment of *Sgs3* sequences with translation in the EG population (Cairo) of *D. melanogaster*. (PDF 1733 kb)

Additional file 5: **Figure S4.** Partial alignment of *Sgs4* sequences with translation in *D. melanogaster* individuals. EF: Ethiopia; Chavroche and Gally: France; Cotonou: Benin; Delhi: India; Cayenne: French Guyana; Tai: Ivory Coast. (PDF 2312 kb)

Additional file 6: **Figure S5.** Partial alignment of Sgs4 protein sequences in the EG population (Cairo) and ZI (Zambia) of *D. melanogaster*. The reference sequence is shown. Asterisks indicate premature stop codons. (PDF 966 kb)

Additional file 7: **Figure S6.** Partial alignment of Sgs3 (A) and Sgs4 (B) amino acid sequences in *D. mauritiana* individuals. Sgs3 mau and Sgs4 mau are the sequences from the online genome. Sgs4 mau has been corrected with our resequencing. Xs are undetermined amino acids. (PPTX 452 kb)

Additional file 8: **Figure S7.** Output trees of Branch-Site-REL analyses (classic.datamonkey.org). “The hue of each color indicates strength of selection, with primary red corresponding to ω>5, primary blue to w = 0 and grey to w = 1. The width of each color component represent the proportion of sites in the corresponding class. Thicker branches have been classified as undergoing episodic diversifying selection by the sequential likelihood ratio test at corrected *p* ≤ 0.05”. MEL: melanogaster, SIM: simulans, SECH: sechellia, SAN: santomea, YAK: yakuba, ERE: erecta, TAK: takahashii, SUZ: suzukii, BIAR: biarmipes, FIC: ficusphila, KIK: kikkawai, ANA: ananassae, BIP: bipectinata. (PDF 51 kb)

Additional file 9: **Table S2.** List of primers used for this study. Different combinations were used to amplify glue genes. All primers were chosen outside the repeated regions. *D. sechellia*, *D. santomea*, *D. virilis* and *D. biarmipes* were resequenced because of uncertainties or putative errors in the online sequences. *D. melanogaster* and *D. mauritiana* were resequenced for studying RNV in *Sgs3* and *Sgs4*. (DOCX 102 kb)

Additional file 10: **Table S3.** Assembly/Annotation error estimation and gene gain/loss rates in a single *λ* model in the 25 *Drosophlia* species included in this study compared to previous studies using fewer species. (DOCX 48 kb)

Additional file 11: **Table S4.** Summary of gene gain and loss events inferred after correcting for annotation and assembly error across all 25 *Drosophila* species. The number of rapidly evolving families is shown in parentheses for each type of change. (DOCX 107 kb)
