# Supplementary figures and images for: Evolution of salivary glue genes in Drosophila species
Source: BMC Evol Biol. 2019 Jan 29;19:36. doi: 10.1186/s12862-019-1364-9 (PMC6352337; doi:10.1186/s12862-019-1364-9)

3 - Sgs1-3-7-8

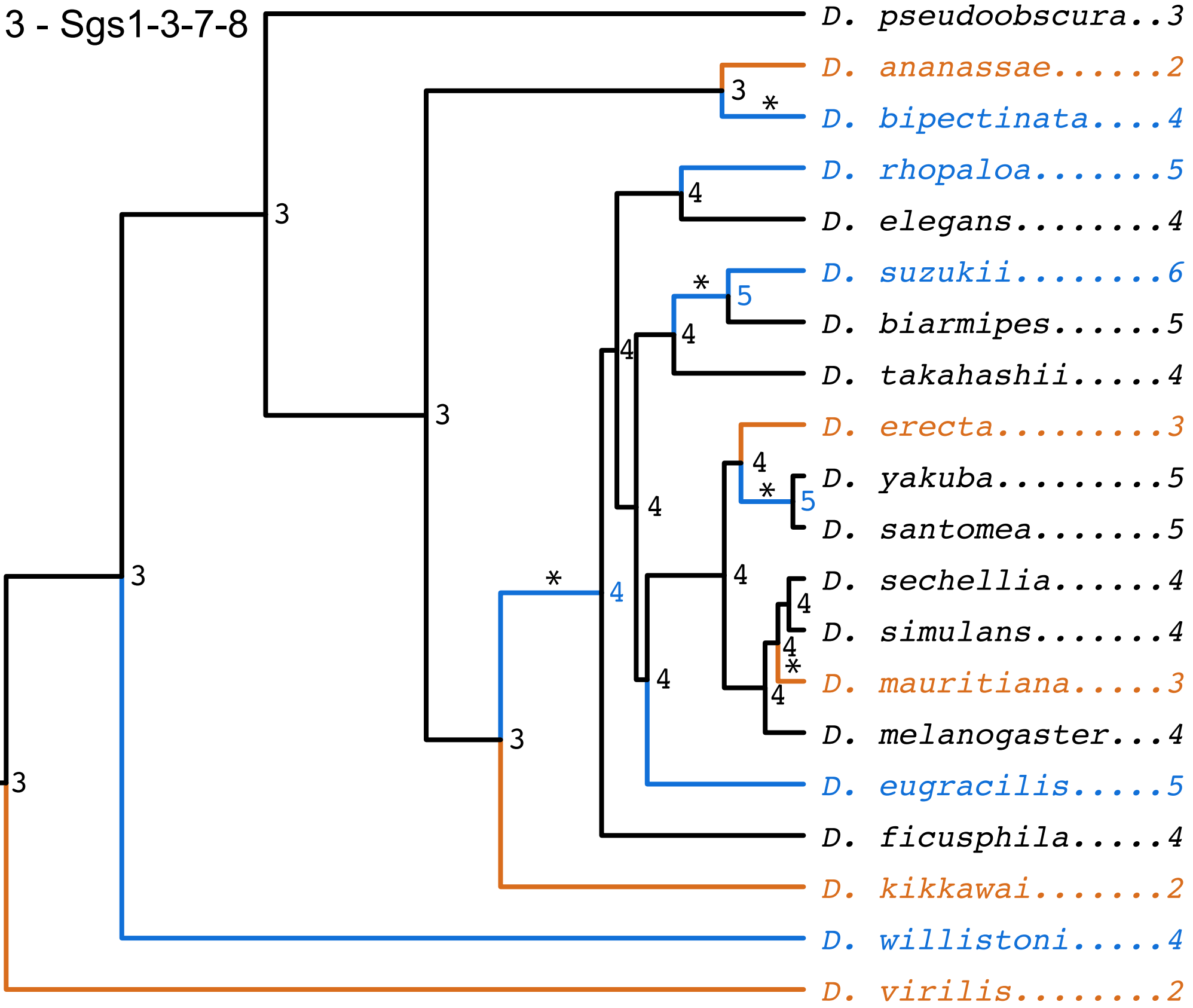

Supplement: Supplementary file 2 — Figure S1. Ancestral states for the Sgs1–3–7-8 gene family inferred by CAFE. Species tips are labeled with the observed gene count and internal nodes are labeled with inferred gene counts. Orange branches represent gene losses, blue branches represent gene gains, while black branches represent lineages in which no change in gene copy number is observed. Branches marked with asterisks have marginally significant p-values (< 0.05). (PDF 173 kb) [file 12862_2019_1364_MOESM2_ESM.pdf]

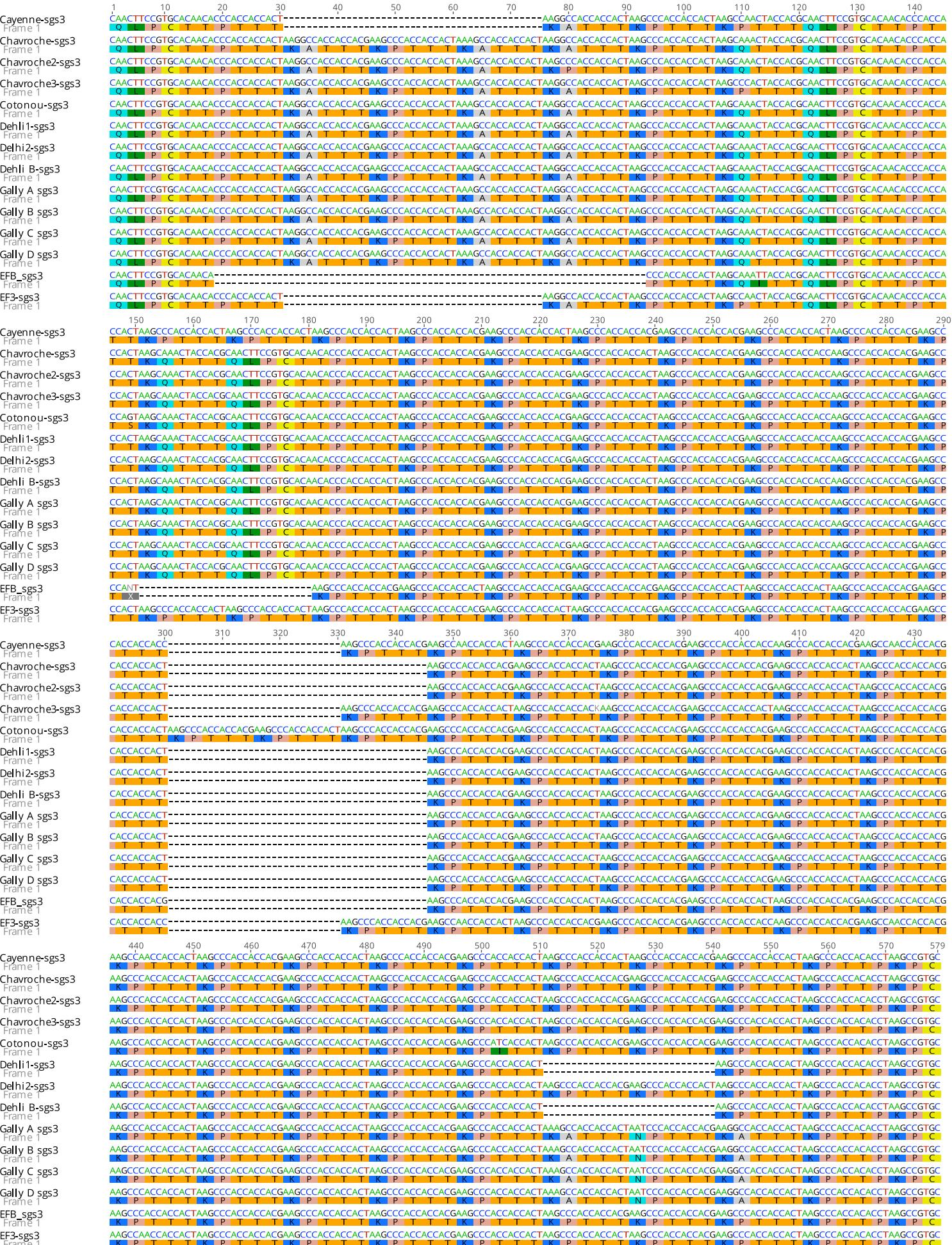

Supplement: Supplementary file 3 — Figure S2. Partial alignment of Sgs3 sequences with translation in D. melanogaster individuals. EF: Ethiopia; Chavroche and Gally: France; Cotonou: Benin; Delhi: India; Cayenne: French Guyana. (PDF 2258 kb) [file 12862_2019_1364_MOESM3_ESM.pdf]

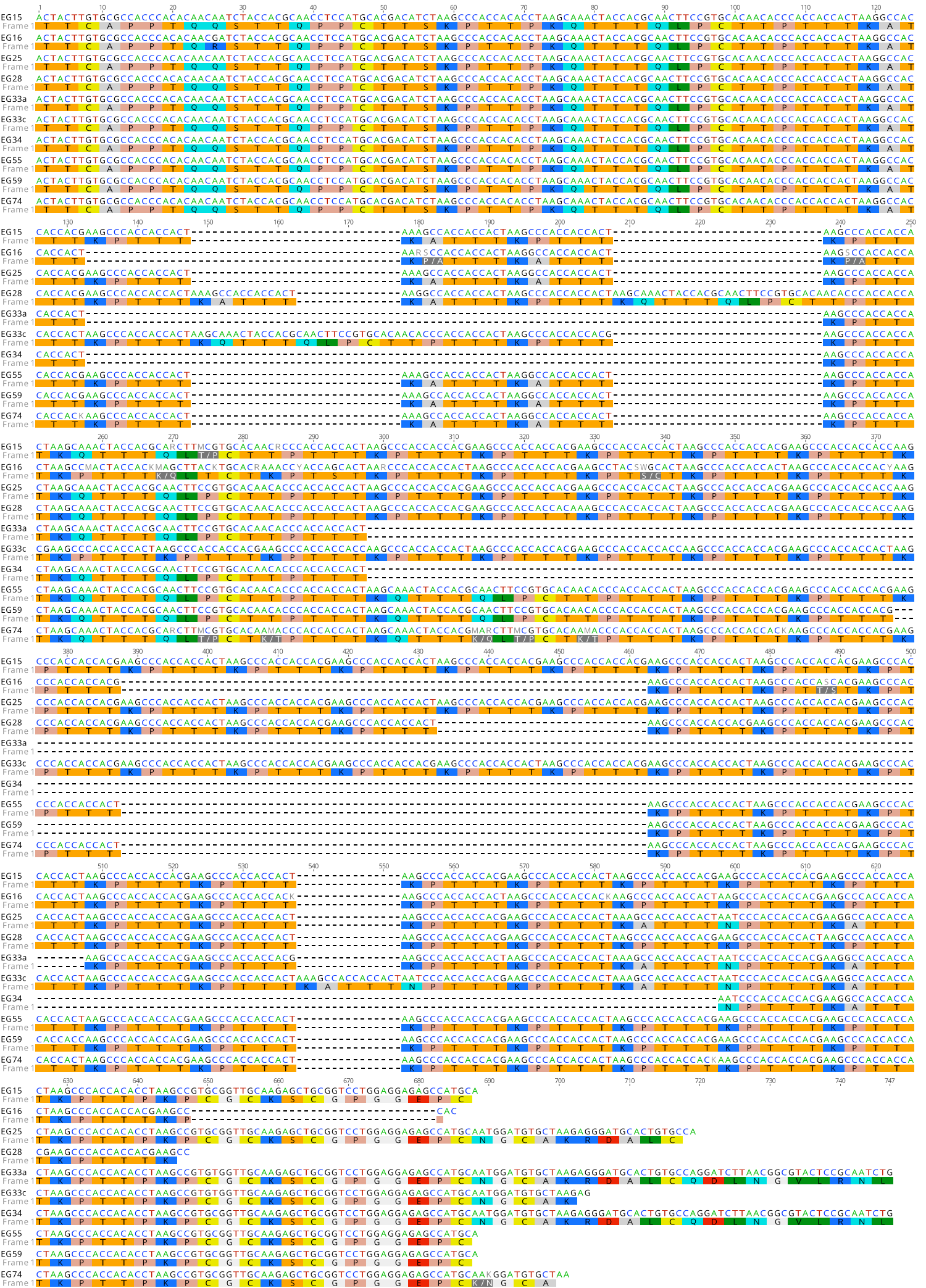

Supplement: Supplementary file 4 — Figure S3. Partial alignment of Sgs3 sequences with translation in the EG population (Cairo) of D. melanogaster. (PDF 1733 kb) [file 12862_2019_1364_MOESM4_ESM.pdf]

## Slide 1
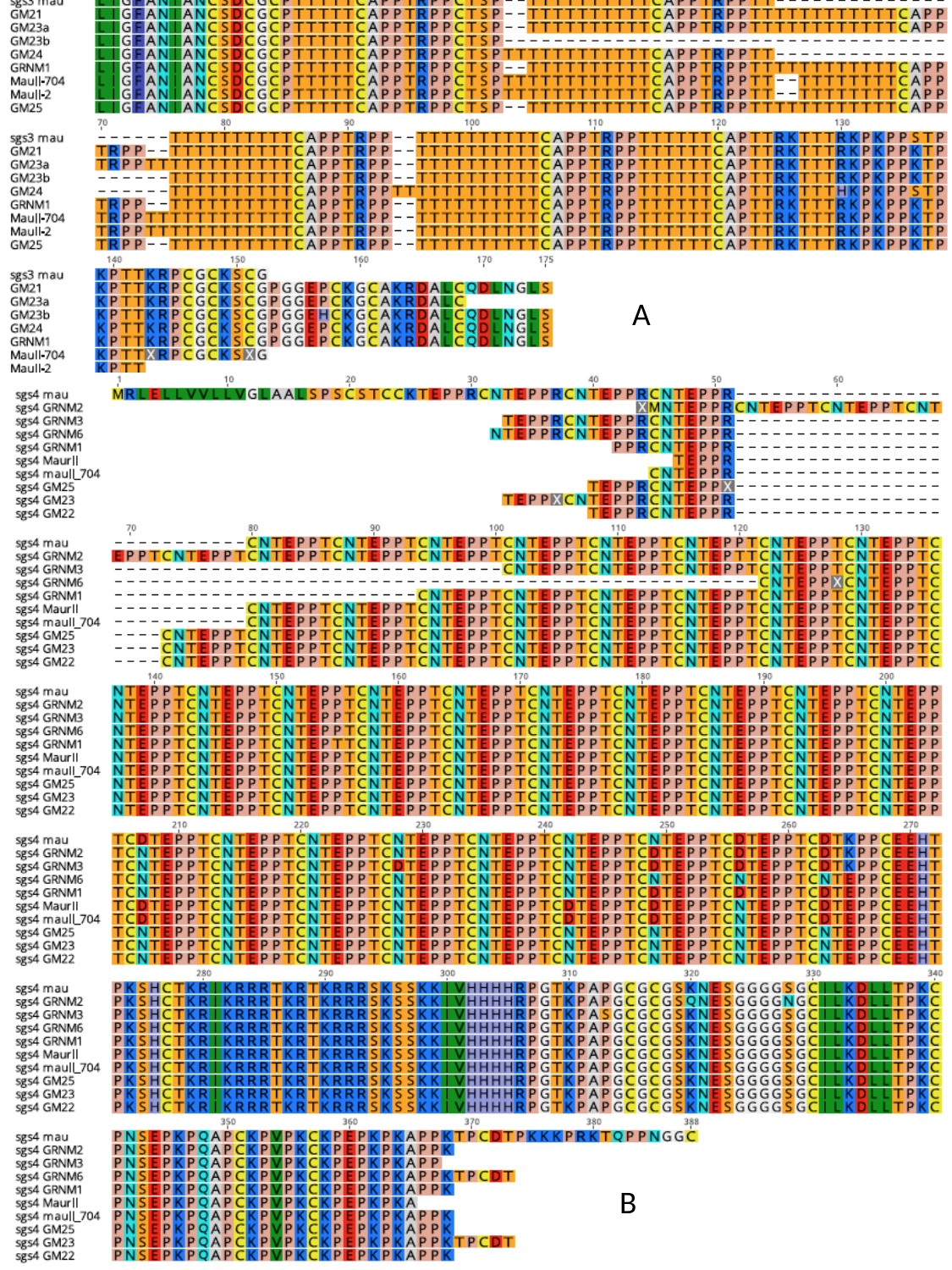

A
B

Supplement: Supplementary file 7 — Figure S6. Partial alignment of Sgs3 (A) and Sgs4 (B) amino acid sequences in D. mauritiana individuals. Sgs3 mau and Sgs4 mau are the sequences from the online genome. Sgs4 mau has been corrected with our resequencing. Xs are undetermined amino acids. (PPTX 452 kb) [file 12862_2019_1364_MOESM7_ESM.pptx]

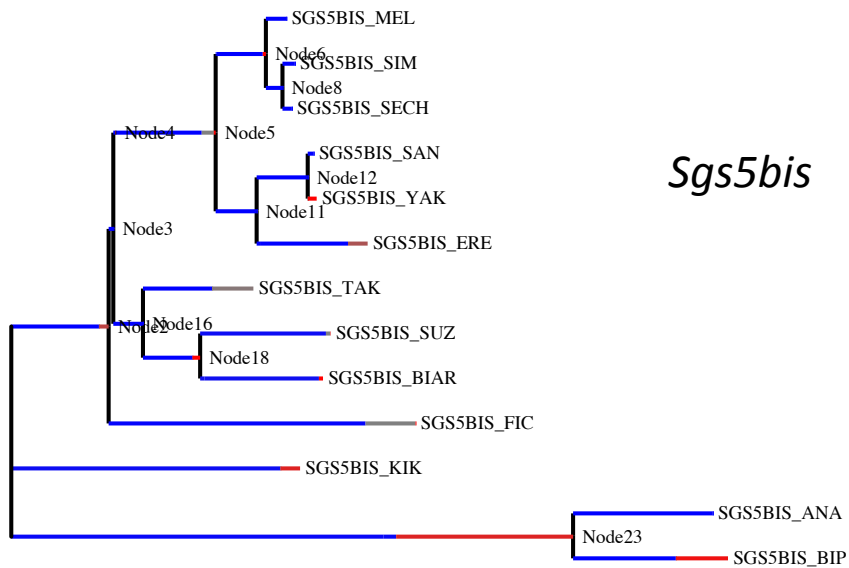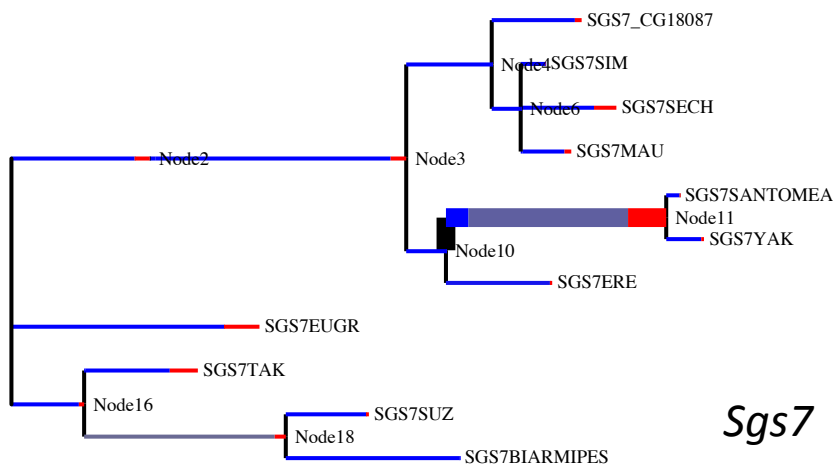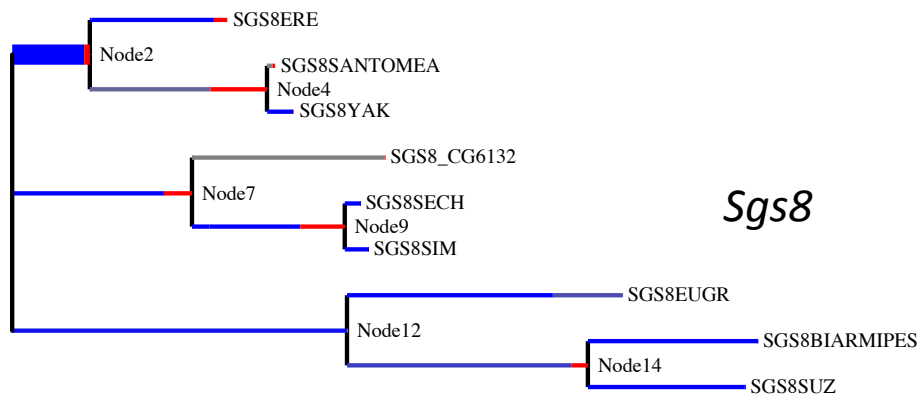

Supplement: Supplementary file 8 — Figure S7. Output trees of Branch-Site-REL analyses (classic.datamonkey.org). “The hue of each color indicates strength of selection, with primary red corresponding to ω>5, primary blue to w = 0 and grey to w = 1. The width of each color component represent the proportion of sites in the corresponding class. Thicker branches have been classified as undergoing episodic diversifying selection by the sequential likelihood ratio test at corrected p ≤ 0.05”. MEL: melanogaster, SIM: simulans, SECH: sechellia, SAN: santomea, YAK: yakuba, ERE: erecta, TAK: takahashii, SUZ: suzukii, BIAR: biarmipes, FIC: ficusphila, KIK: kikkawai, ANA: ananassae, BIP: bipectinata. (PDF 51 kb) [file 12862_2019_1364_MOESM8_ESM.pdf]
