# Supplementary material for: Evolution of salivary glue genes in Drosophila species
Source: BMC Evol Biol. 2019 Jan 29;19:36. doi: 10.1186/s12862-019-1364-9 (PMC6352337; doi:10.1186/s12862-019-1364-9)
Supplement: Supplementary file 6 — Figure S5. Partial alignment of Sgs4 protein sequences in the EG population (Cairo) and ZI (Zambia) of D. melanogaster. The reference sequence is shown. Asterisks indicate premature stop codons. (PDF 966 kb) [file 12862_2019_1364_MOESM6_ESM.pdf]

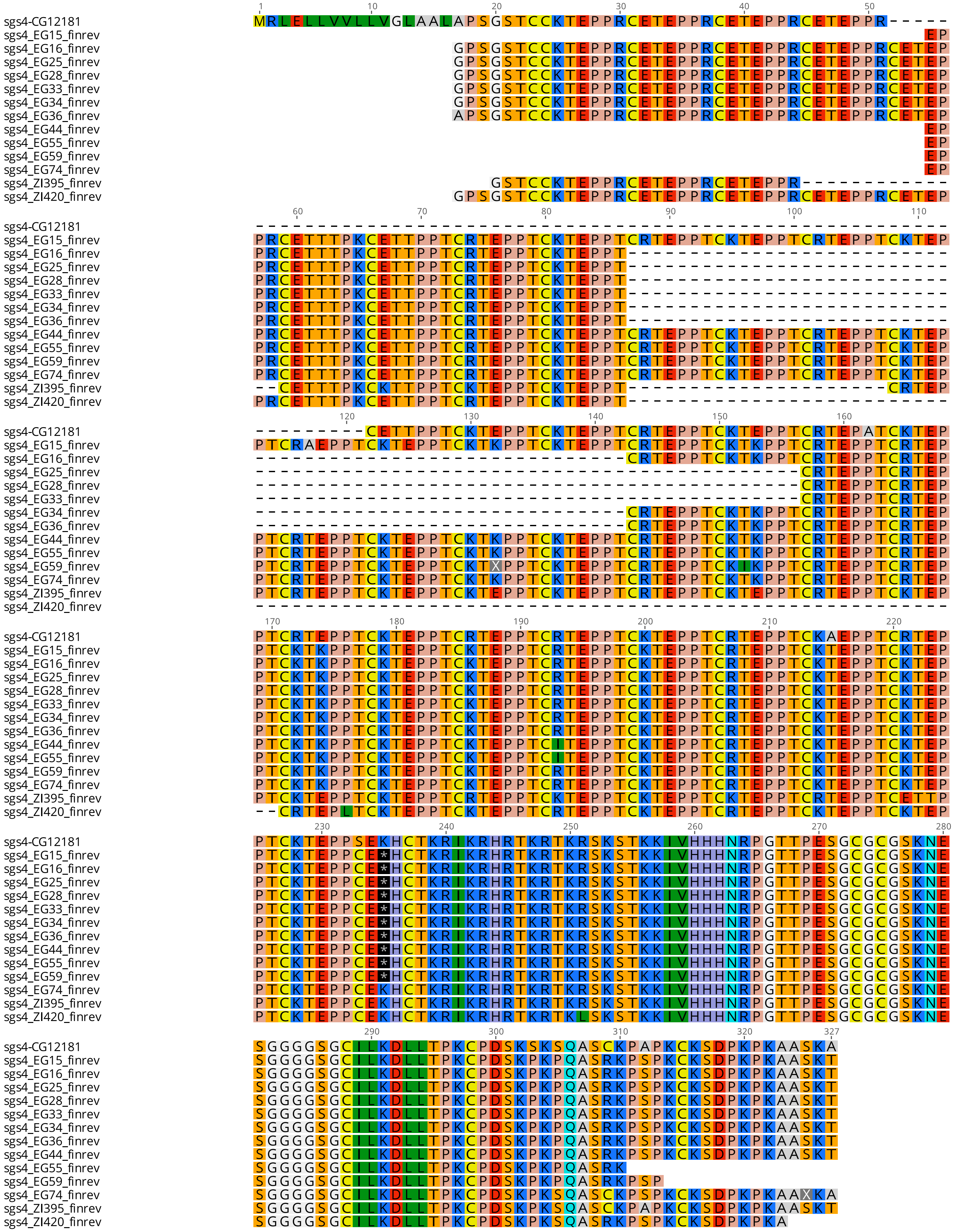
sgs4-CG12181
sgs4_EG15_finrev
sgs4_EG16_finrev
sgs4_EG25_finrev
sgs4_EG28_finrev
sgs4_EG33_finrev
sgs4_EG34_finrev
sgs4_EG36_finrev
sgs4_EG44_finrev
sgs4_EG55_finrev
sgs4_EG59_finrev
sgs4_EG74_finrev
sgs4_ZI395_finrev
sgs4_ZI420_finrev
1
10
20
30
40
50
60
70
80
90
100
110
120
130
140
150
160
170
180
190
200
210
220
230
240
250
260
270
280
290
300
310
320
327
