# Supplementary material for: Evolution of salivary glue genes in Drosophila species
Source: BMC Evol Biol. 2019 Jan 29;19:36. doi: 10.1186/s12862-019-1364-9 (PMC6352337; doi:10.1186/s12862-019-1364-9)
Supplement: Supplementary file 9 — Table S2. List of primers used for this study. Different combinations were used to amplify glue genes. All primers were chosen outside the repeated regions. D. sechellia, D. santomea, D. virilis and D. biarmipes were resequenced because of uncertainties or putative errors in the online sequences. D. melanogaster and D. mauritiana were resequenced for studying RNV in Sgs3 and Sgs4. (DOCX 102 kb) [file 12862_2019_1364_MOESM9_ESM.docx]

**Table S2 :** List of primers used for this study. Different combinations were used to amplify glue genes. All primers were chosen outside the repeated regions. *D. sechellia*, *D. santomea*, *D. virilis* and *D. biarmipes* were resequenced because of uncertainties or putative errors in the online sequences. *D. melanogaster* and *D. mauritiana* were resequenced for studying RNV in *Sgs3* and *Sgs4*, or confirming the presence of premature stop codons in *Sgs4* and *Sgs5* of *D. melanogaster*.

***Sgs1***

| species | Forward primers | Reverse primers |
| --- | --- | --- |
| *D. melanogaster* | ATGAAAGTCGCCCTTATCTT | TGTCAGTGTCACAGGGTTGC |
| *D. sechellia* | TCGTCCTTGTCCTTGCCATC | TTACGTTCGTGTGCACAAGCA |

***Sgs3***

| species | Forward primers | Reverse primers |
| --- | --- | --- |
| *D. melanogaster* | TCCGCTAATGTTGCCAACTG  TAATGTTGCCAACTGTTGC  CATGAAGCTGACCATTGCTA | CATCCATTGCATGGCTCTCC  ATTGACGGATCTTGCGCTC  GACGCATTGACGGATCTTGC |
| *D. sechellia* | GCTGACCATTGCTACCATTG | CTTAGCGCATCCTGTGCAAG |
| *D. santomea* | GAAGCTGACCATTGCCATCGC  GTGCACAGTCCCTGCCTCTT | TAAGATCCGTGCACAGTCCC |
| *D. mauritiana* | ATCCTGCTTATTGGCTTCGC | AAGCCGTTGAGATCCTGGCA |
| *D. virilis* | TGCTCGGCTGAATGCGAGTG | CAAGAGCTCCTTGCAGAGCG  GGTCCTTGTGGTGGACACGC |
| *D. biarmipes* | AGCCCATGTGAAGTGGAGTC  GGATGCACCACCGGGACTGA  ACCGGGACTGAATGTGGTTG  TGGAGTCTCCACCCTGTGA | GGGCTCTCCGCAGACGCATTG  CATTGCCGGATCTTGCGTTC  ACAAACGCACTGGCGGATTC  TCCAGACCTCGGACTGTGTTG |

***Sgs4***

| species | Forward primers | Reverse primers |
| --- | --- | --- |
| *D. melanogaster* | CACTTGCCCCGTCAGGTTCTAC  TGTCTATGTCTAACCATCGC | TACAGCATGTAGAACCTGAC  GCGGTTTCTTGGTGGTGTTC  TGTCGCTTGATGCGTTTCGT |
| *D. sechellia* | GGTCTGGCTGCACTTTCGCC | TACAGCATGTAGAACCTGAC |
| *D. santomea* | TTATTGGTCGGGTTGGCTG  TCGGGTTGGCTGCACTTAC | GGTGGTGGCACATGGTTTCG  CACATGGTTTCGGCTGCGA |
| *D. mauritiana* | CTGCACTTTCGCCGTCATGT | CTGGGTCTTCCGTGGTTTC |

***Sgs5***

| species | Forward primers | Reverse primers |
| --- | --- | --- |
| *D. melanogaster* | CATGGACAAGCCGTCCAGGA | GTAACGTGATCCAACTGAATCC |
