# Supplementary material for: Evolution of salivary glue genes in Drosophila species
Source: BMC Evol Biol. 2019 Jan 29;19:36. doi: 10.1186/s12862-019-1364-9 (PMC6352337; doi:10.1186/s12862-019-1364-9)
Supplement: Supplementary file 10 — Table S3. Assembly/Annotation error estimation and gene gain/loss rates in a single λ model in the 25 Drosophlia species included in this study compared to previous studies using fewer species. (DOCX 48 kb) [file 12862_2019_1364_MOESM10_ESM.docx]

Table S3: Assembly/Annotation error estimation and gene gain/loss rates in a single *λ* model in the 25 *Drosophlia* species included in this study compared to previous studies using fewer species.

|  | ***λ*** (No Error Model) | ***ε*** (Estimated error) | ***λ*** (Error Model = ***ε***) |
| --- | --- | --- | --- |
| 25 *Drosophila* species in this study | 0.00365 | 0.04434 | 0.00340 |
| 12 *Drosophila* species (Han et al. 2013) | 0.00121 | 0.04102 | 0.00059 |
| 12 *Drosophila* species (Hahn et al. 2007) | 0.00121 | NA | NA |
