# Supplementary material for: Evolution of salivary glue genes in Drosophila species
Source: BMC Evol Biol. 2019 Jan 29;19:36. doi: 10.1186/s12862-019-1364-9 (PMC6352337; doi:10.1186/s12862-019-1364-9)
Supplement: Supplementary file 11 — Table S4. Summary of gene gain and loss events inferred after correcting for annotation and assembly error across all 25 Drosophila species. The number of rapidly evolving families is shown in parentheses for each type of change. (DOCX 107 kb) [file 12862_2019_1364_MOESM11_ESM.docx]

Table S4: Summary of gene gain and loss events inferred after correcting for annotation and assembly error across all 25 *Drosophila* species. The number of rapidly evolving families is shown in parentheses for each type of change.

| Species | Expansions |  |  | Contractions |  |  | No Change | Avg. Expansion |
| --- | --- | --- | --- | --- | --- | --- | --- | --- |
|  | Families | Genes gained | genes/  expansion | Families | Genes lost | genes/  contraction |  |  |
| *pseudoobscura* | 307 (55) | 390 | 1.27 | 325 (79) | 356 | 1.1 | 8747 | 0.003625 |
| *miranda* | 161 (14) | 189 | 1.17 | 377 (27) | 428 | 1.14 | 8841 | -0.025482 |
| *bipectinata* | 425 (43) | 579 | 1.36 | 167 (12) | 206 | 1.23 | 8787 | 0.039770 |
| *eugracilis* | 461 (34) | 649 | 1.41 | 271 (6) | 311 | 1.15 | 8647 | 0.036038 |
| *busckii* | 844 (23) | 1288 | 1.53 | 712 (5) | 801 | 1.13 | 7823 | 0.051925 |
| *takahashii* | 477 (60) | 874 | 1.83 | 139 (4) | 148 | 1.06 | 8763 | 0.077407 |
| *erecta* | 102 (20) | 132 | 1.29 | 198 (25) | 286 | 1.44 | 9079 | -0.016420 |
| *sechellia* | 1184 (233) | 2020 | 1.71 | 443 (83) | 480 | 1.08 | 7752 | 0.164197 |
| *persimilis* | 1243 (254) | 1979 | 1.59 | 458 (66) | 493 | 1.08 | 7678 | 0.158439 |
| *kikkawai* | 558 (30) | 811 | 1.45 | 362 (4) | 410 | 1.13 | 8459 | 0.042755 |
| *arizonae* | 154 (31) | 163 | 1.06 | 531 (106) | 578 | 1.09 | 8694 | -0.044248 |
| *virilis* | 329 (21) | 541 | 1.64 | 288 (1) | 303 | 1.05 | 8762 | 0.025376 |
| *elegans* | 347 (24) | 464 | 1.34 | 259 (24) | 357 | 1.38 | 8773 | 0.011408 |
| *suzukii* | 1472 (105) | 2108 | 1.43 | 219 (8) | 238 | 1.09 | 7688 | 0.199382 |
| *simulans* | 151 (35) | 192 | 1.27 | 168 (66) | 260 | 1.55 | 9060 | -0.007250 |
| *yakuba* | 673 (84) | 891 | 1.32 | 120 (6) | 145 | 1.21 | 8586 | 0.079539 |
| *melanogaster* | 60 (13) | 88 | 1.47 | 166 (13) | 196 | 1.18 | 9153 | -0.011515 |
| *mojavensis* | 320 (101) | 470 | 1.47 | 166 (47) | 179 | 1.08 | 8893 | 0.031027 |
| *grimshawi* | 843 (25) | 1218 | 1.44 | 633 (2) | 683 | 1.08 | 7903 | 0.057042 |
| *willistoni* | 603 (13) | 895 | 1.48 | 649 (1) | 713 | 1.1 | 8127 | 0.019405 |
| *navojoa* | 202 (25) | 227 | 1.12 | 1934 (74) | 2200 | 1.14 | 7243 | -0.210364 |
| *rhopaloa* | 1127 (96) | 1753 | 1.56 | 153 (2) | 159 | 1.04 | 8099 | 0.169954 |
| *biarmipes* | 150 (9) | 184 | 1.23 | 230 (21) | 288 | 1.25 | 8999 | -0.011089 |
| *ananassae* | 270 (28) | 464 | 1.72 | 237 (13) | 278 | 1.17 | 8872 | 0.019832 |
| *ficusphila* | 514 (29) | 685 | 1.33 | 341 (7) | 393 | 1.15 | 8524 | 0.031133 |
