# Supplementary material for: Evolution of salivary glue genes in Drosophila species
Source: BMC Evol Biol. 2019 Jan 29;19:36. doi: 10.1186/s12862-019-1364-9 (PMC6352337; doi:10.1186/s12862-019-1364-9)
Supplement: Supplementary file 5 — Figure S4. Partial alignment of Sgs4 sequences with translation in D. melanogaster individuals. EF: Ethiopia; Chavroche and Gally: France; Cotonou: Benin; Delhi: India; Cayenne: French Guyana; Tai: Ivory Coast. (PDF 2312 kb) [file 12862_2019_1364_MOESM5_ESM.pdf]

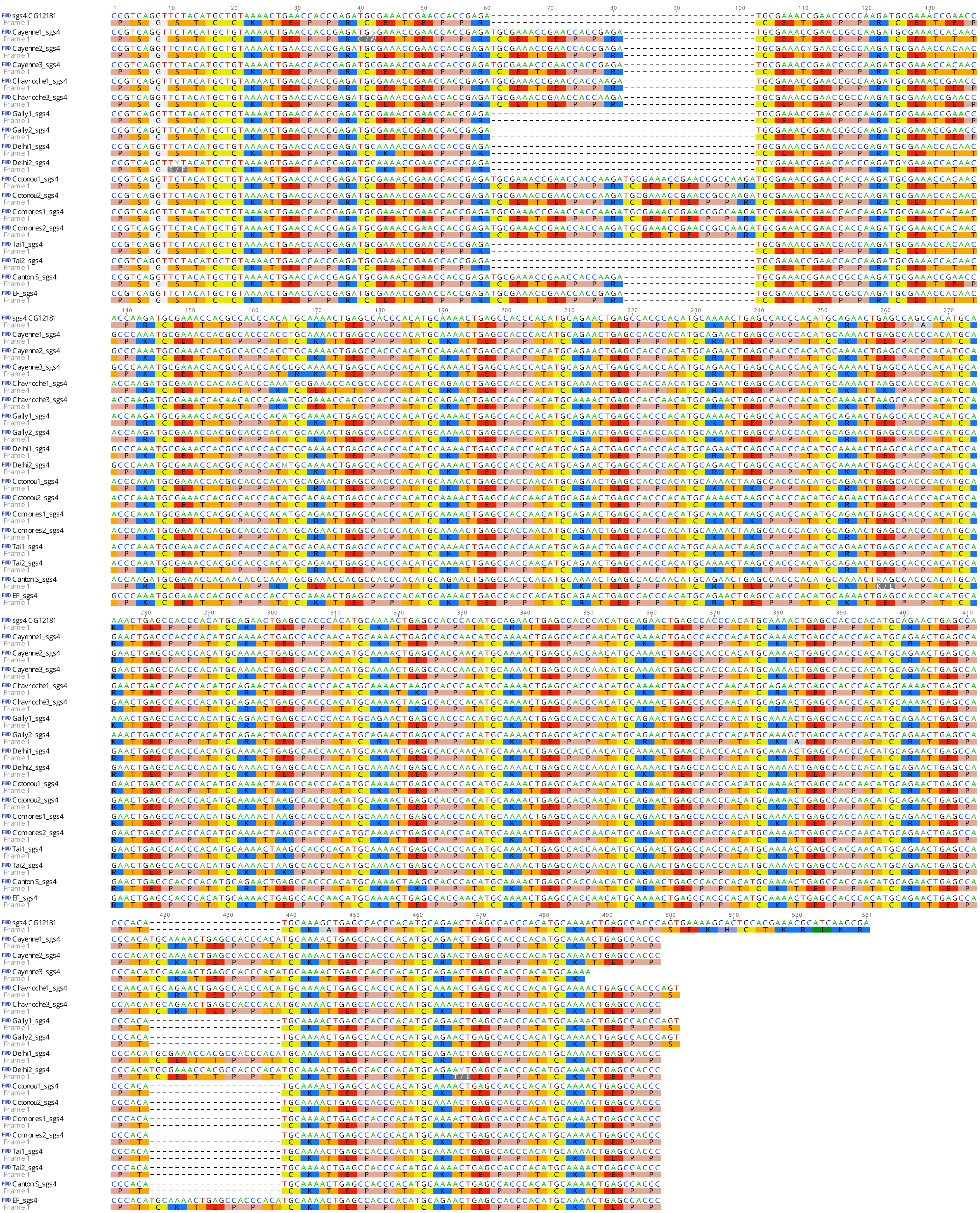
sgs4 CG12181
Cayenne1_sgs4
Cayenne2_sgs4
Cayenne3_sgs4
Chavroche1_sgs4
Chavroche3_sgs4
Gally1_sgs4
Gally2_sgs4
Delhi1_sgs4
Delhi2_sgs4
Cotonou1_sgs4
Cotonou2_sgs4
Comores1_sgs4
Comores2_sgs4
Tai1_sgs4
Tai2_sgs4
Canton S_sgs4
EF_sgs4
